# The global burden of aortic aneurysm in adults over 55: evolving trends, risk factors, and projections

**DOI:** 10.3389/fcvm.2025.1629635

**Published:** 2025-11-03

**Authors:** Guoqian Ma, Yuan Li, Fan Jia

**Affiliations:** ^1^ Department of Cardiology, Key Laboratory of Cardiovascular Disease of Yunnan Province, Clinical Medicine Center for Cardiovascular Disease of Yunnan Province, Yan'an Affiliated Hospital of Kunming Medical University, Kunming, China; ^2^ Ultrasound Department, Kunming Maternal and Child Health Hospital, Kunming, China

**Keywords:** aortic aneurysm (AA), disability-adjusted life years (DALYs), global burden of disease (GBD), Bayesian age-period-cohort (BAPC), mortality

## Abstract

**Background:**

Aortic aneurysm (AA) is a life-threatening vascular disease and a major contributor to global cardiovascular mortality, particularly in older adults. This study aims to report global trends in mortality and disability-adjusted life years (DALYs) attributable to AA among adults aged 55 years and older from 1990 to 2021.

**Methods:**

We conducted a comprehensive analysis of aortic aneurysm mortality and disability-adjusted life years (DALYs) from 1990 to 2021, utilizing data from the Global Burden of Disease (GBD) study. Trends were examined by sex, age group, socio-demographic index (SDI), region, and country. Joinpoint regression was used to assess annual percentage changes (APCs), and Bayesian age-period-cohort (BAPC) modeling projected AA burden to 2035. Primary risk factors were also analyzed.

**Results:**

Between 1990 and 2021, global AA-related deaths rose by 73.9% [from 79,608 (95% UI, 74,398–84,032) to 138,450 (123,754–149,214)], while the mortality rate declined by 21.4% (from 11.86 to 9.32 per 100,000). Similar patterns were observed for DALYs, with a 62.6% increase in total DALYs but a 26.5% decrease in rates. Males and older adults experienced disproportionately higher mortality and DALY rates. Marked regional and national disparities emerged: the greatest increases in AA burden were seen in low-middle SDI regions [mortality rate EAPC, 1.49 [95% CI, 1.43–1.54]; DALY rate EAPC, 1.29 [95% CI, 1.23–1.35]], while high-income North America and Australasia achieved the largest reductions. The leading risk factor globally was smoking, particularly among males. BAPC projections indicate continued declines in age-standardized AA mortality and DALY rates through 2035, though absolute numbers will likely remain high.

**Interpretation:**

Although rates of AA-related mortality and DALYs have declined globally, the absolute burden continues to rise, driven by population aging and persistent risk factors. Disparities across regions and SDI groups highlight the urgent need for targeted prevention, including tobacco control, risk factor management, and selective screening—especially in rapidly aging, low-resource settings. Strengthening health system capacity for both prevention and surgical intervention will be critical to curbing future AA burden.

## Introduction

Aortic aneurysm (AA) is a chronic disease of the aorta characterized by irreversible, localized dilation resulting from maladaptive remodeling of the aortic wall. This condition can progress to life-threatening aortic rupture, which carries a mortality rate exceeding 80% and is responsible for an estimated 150,000–200,000 deaths worldwide each year (1–3). Aneurysms can occur in any segment of the aorta, most commonly in the abdominal aorta (abdominal aortic aneurysm, AAA) or the thoracic aorta (thoracic aortic aneurysm, TAA) (4). The pathophysiology involves degeneration of the aortic wall connective tissue, inflammation, and biomechanical stress leading to progressive weakening and expansion of the vessel. AAA is far more frequent than TAA in the general population and is strongly linked to atherosclerosis, whereas TAA may be associated with genetic syndromes (e.g., Marfan, Ehlers-Danlos) or uncontrolled hypertension (5–7). Importantly, most aortic aneurysms remain asymptomatic (“silent”) until they enlarge or rupture (8). Aortic aneurysms typically remain asymptomatic until rupture, making diagnosis and clinical management particularly difficult; an estimated 1.5 million Americans had undiagnosed abdominal aortic aneurysms in 2003 (9).

Multiple risk factors contribute to the development and progression of aortic aneurysms. Aneurysms rarely occur before age 50, so advancing age is a key non-modifiable risk factor; moreover, their prevalence rises sharply in the elderly (10). Sex plays a role, with men having a substantially higher prevalence and risk of AAA and tending to develop aneurysms at younger ages (11). Smoking is the most important modifiable risk factor; both current and former smokers have markedly elevated AAA risk, with smoking attributed as a causal factor in ∼30%–60% of aneurysm-related deaths in various populations (12). Other modifiable factors include hypertension and high systolic blood pressure, which especially contribute to TAA and aneurysm rupture risk (particularly in women) (13). Elevated cholesterol and a history of other atherosclerotic cardiovascular diseases are also associated with aneurysm formation (14). In light of these risk profiles, screening programs (e.g., one-time ultrasound screening for AAA in older male smokers) have been implemented in some high-income countries to facilitate early detection and elective repair, aiming to prevent rupture (15).

Given the constantly changing global population structure and risk factor profiles, understanding the global burden of aortic aneurysm is crucial for public health planning. Previous Global Burden of Disease (GBD) analyses have provided insights up to 2017 and 2019 (16, 17). In this context, this study utilizes the GBD database to analyze and statistically report the incidence, mortality, and disability-adjusted life years (DALYs) of AA among adults aged 55 and older from 1990 to 2021. The findings of this study aim to provide data support for the development of new diagnostic and treatment strategies globally, ultimately aiming to reduce the worldwide burden of this disease.

## Methods

### Data sources

This study was approved by the Ethics Committee of Kunming Yan'an Hospital, which granted a waiver of informed consent on the grounds that only de-identified, aggregated data were analyzed, with no personally identifiable information involved. Data for individuals aged 55 years and older with AA were extracted from the Global Health Data Exchange query tool, developed by the GBD collaborators, which provides standardized disease definitions. The GBD analytical framework enables comparative assessments of mortality across countries, regions, and the globe. Two core indicators were used to quantify disease burden: mortality and DALYs. DALYs are calculated as the sum of years of life lost due to premature mortality (YLL) and years lived with disability (YLD) (18), with the following formulas:

YLL=numberofdeaths×standardlifeexpectancyatageofdeath

YLD=prevalence×disabilityweight

Analyses were stratified by sex, age group (in 5-year intervals for those aged ≥55 years), and geographical location. The study followed the Strengthening the Reporting of Observational Studies in Epidemiology (STROBE) guidelines for observational research (19).

### Sociodemographic index

The sociodemographic index (SDI) is a composite metric reflecting development based on fertility rates, educational attainment, and per capita income. SDI values range from 0 to 1, with higher values indicating greater socioeconomic development (20). SDI has been shown to correlate with disease incidence and mortality. In this study, countries and regions were classified into five SDI quintiles (low, low-middle, middle, high-middle, and high) to explore the relationship between SDI and the burden of aortic aneurysm among adults aged 55 years and older.

### Statistical analysis

Mortality and DALY rates per 100,000 population, along with their 95% uncertainty intervals (UI), were calculated according to GBD protocols. Trends over time were assessed using the Joinpoint regression model, which estimates annual percentage change (APC) and corresponding 95% confidence intervals (CI) to capture period-specific trends (21). This approach provides detailed insights into year-to-year variability. The average estimated annual percentage change (EAPC) and its CI were calculated using a log-linear regression model to quantify long-term trends in mortality and DALYs from 1990 to 2021 among adults aged ≥55 years (22). EAPC is particularly valuable for evaluating overall temporal trends, as it reflects whether rates have generally increased or decreased over the study period, irrespective of short-term fluctuations. An EAPC with a lower 95% CI bound greater than zero indicates a significant upward trend, whereas an upper 95% CI bound below zero denotes a significant downward trend. Associations between disease burden metrics and SDI were examined using curve fitting. All analyses were conducted using R software, version 4.4.2, with statistical significance set at *p* < 0.05.

## Results

### Global burden trends

#### Mortality

**Figure 1:**
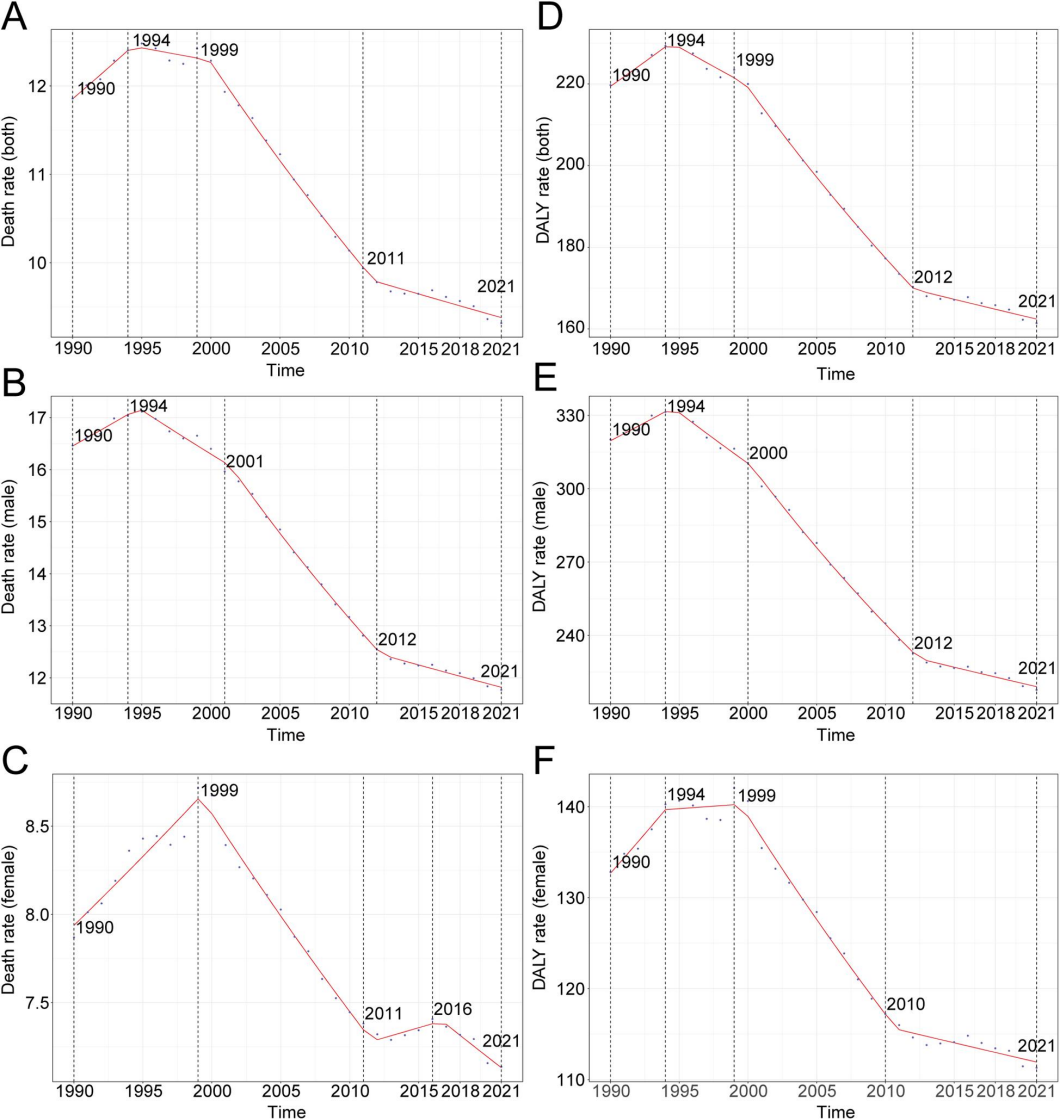
Annual percent change (APC) and trends in global aortic aneurysm mortality and disability-adjusted life years (DALYs) from 1990 to 2021. **(A)** Death rate (both sexes); **(B)** death rate (males); **(C)** death rate (females); **(D)** DALY rate (both sexes); **(E)** DALY rate (males); **(F)** DALY rate (females).

**Figure 2:**
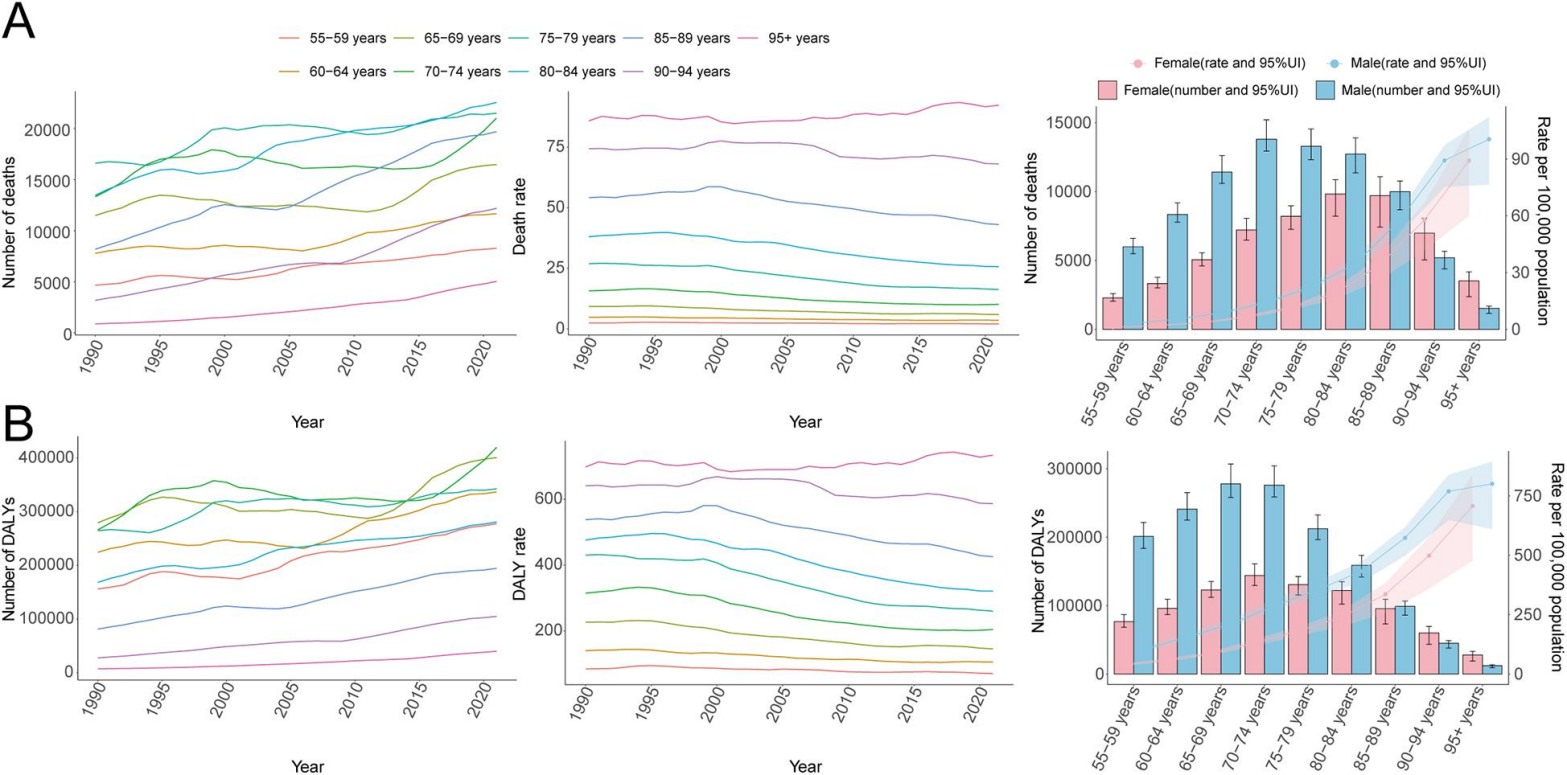
Trends in mortality and disability-adjusted life years (DALYs) of aortic aneurysm by age and sex, 1990–2021. **(A)** Mortality cases and rates. **(B)** DALYs cases and rates.

**Figure 3:**
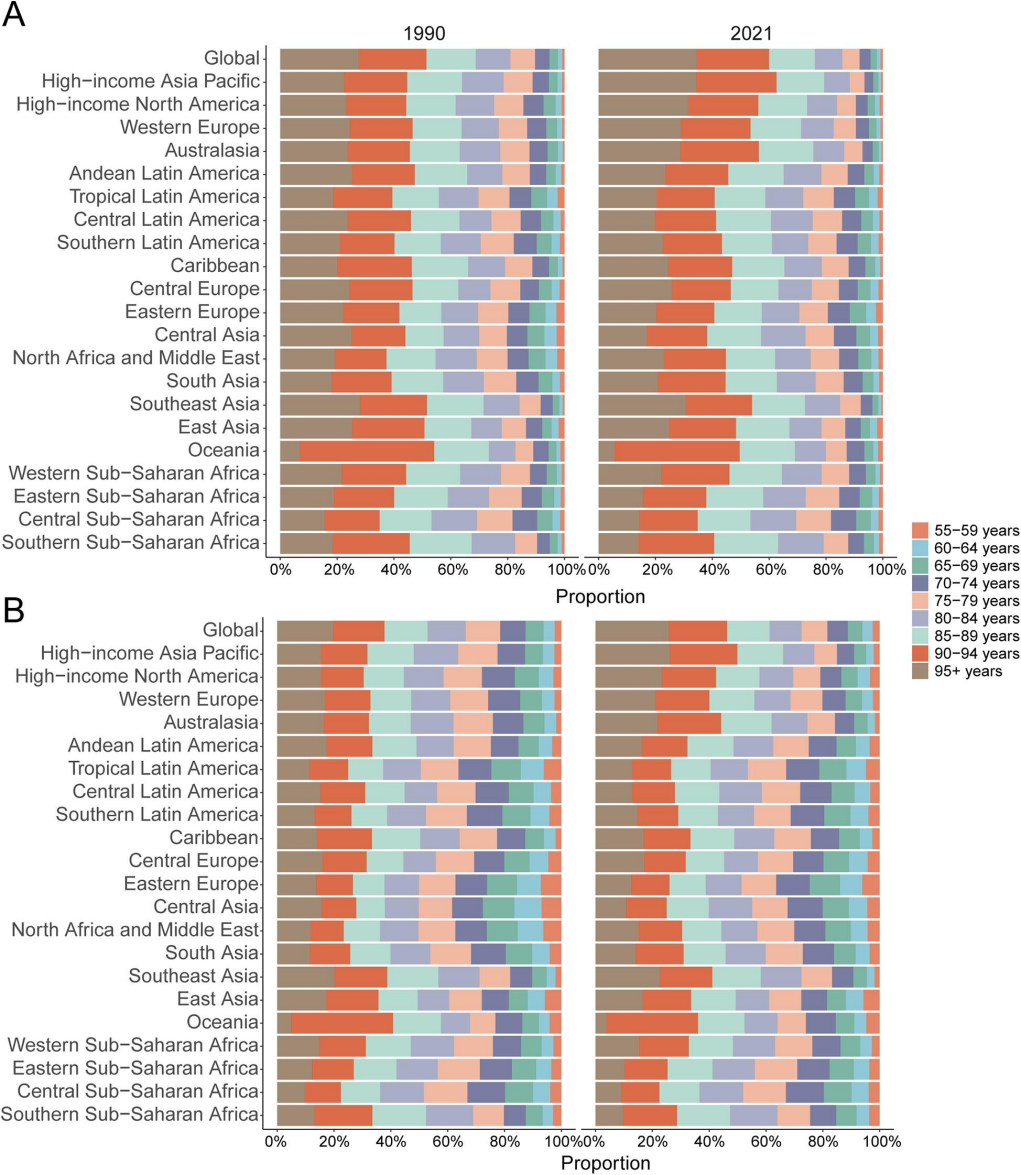
Age-specific percentages of aortic aneurysm mortality and disability-adjusted life years (DALYs) in 2021. **(A)** Deaths. **(B)** DALYs.

Over the past three decades, global AA-related mortality exhibited an initial rise followed by a decline. The highest APC occurred during 1990–1994 at 1.14% (95% CI, 0.80%–1.48%), peaking in 1994 with a mortality rate of 12.40 per 100,000 (95% UI, 11.56–13.08; Figure 1A). Males recorded their highest APC during the same period (0.91%; 95% CI, 0.60%–1.21%), peaking in 1994 at 17.03 per 100,000 (95% UI, 16.05–18.31; Figure 1B), whereas females had the highest APC from 1990 to 1999 (0.97%; 95% CI, 0.81%–1.13%), peaking in 1999 at 8.72 per 100,000 (95% UI, 7.72–9.50; Figure 1C). Total global deaths increased from 79,607.58 (95% UI, 74,397.50–84,032.01) in 1990 to 138,450.22 (95% UI, 123,753.62–149,214.49) in 2021, marking a 73.92% rise (95% UI, 62.85–82.44%; Table 1). Nonetheless, mortality rates decreased overall by 21.42% from 11.86 per 100,000 in 1990 to 9.32 per 100,000 in 2021 (95% UI, 8.33–10.04), with an EAPC of −1.09 (95% CI, −1.22 to −0.95; Table 1). Mortality rates declined across all age groups above 55 except for those over 95, where rates increased by approximately 7.5% (Figure 2A). The highest mortality rate in 2021 was observed among individuals over 95, accounting for 34.5% of AA-related deaths at 92.37 per 100,000 (95% UI, 64.60–107.47), whereas the lowest occurred in those aged 55–59 (2.09 per 100,000; 95% UI, 1.92–2.30), representing only 0.8% of total deaths (Figure 3A). Mortality was consistently higher among males than females across all age groups above 55, particularly between ages 55 and 84 (Figure 2A).

**Table 1:** Mortality of aortic aneurysm between 1990 and 2021 at the global and regional level.

Location	1990 (95% UI)		2021 (95% UI)		1990–2021 (95% UI)		
	Death cases	Death rate	Death cases	Death rate	Cases change^a^	Rate change^a^	EAPC^b^
Global	79,607.58 (74,397.50, 84,032.01)	11.86 (11.08, 12.52)	138,450.22 (123,753.62, 149,214.49)	9.32 (8.33, 10.04)	73.92 (62.85, 82.44)	−21.42 (−26.42, −17.57)	−1.09 (−1.22, −0.95)
High SDI	51,071.57 (47,779.66, 52,698.60)	27.39 (25.62, 28.26)	63,673.55 (54,171.11, 68,784.57)	18.46 (15.70, 19.94)	24.68 (13.49, 31.39)	−32.62 (−38.66, −28.99)	−1.70 (−1.86, −1.54)
High-middle SDI	15,596.83 (14,863.36, 16,247.41)	9.04 (8.62, 9.42)	30,776.97 (28,510.43, 32,911.58)	8.88 (8.22, 9.49)	97.33 (82.26, 111.47)	−1.80 (−9.30, 5.24)	−0.40 (−0.59, −0.20)
Middle SDI	6,900.25 (6,360.25, 7,667.24)	3.98 (3.66, 4.42)	24,295.67 (22,015.17, 26,392.91)	5.17 (4.69, 5.62)	252.10 (211.02, 290.42)	30.06 (14.89, 44.22)	0.60 (0.42, 0.78)
Low-middle SDI	3,794.10 (3,001.84, 5,161.93)	3.76 (2.98, 5.12)	14,323.09 (11,893.41, 19,198.01)	5.94 (4.93, 7.96)	277.51 (213.93, 345.28)	57.84 (31.26, 86.18)	1.49 (1.43, 1.54)
Low SDI	2,125.00 (1,286.46, 3,694.07)	5.70 (3.45, 9.90)	5,204.64 (3,240.03, 8,583.43)	6.34 (3.95, 10.46)	144.92 (100.28, 199.32)	11.36 (−8.94, 36.09)	0.27 (0.09, 0.44)
Regions							
Andean Latin America	156.06 (132.58, 185.43)	4.65 (3.95, 5.53)	460.04 (383.35, 551.60)	4.64 (3.87, 5.57)	194.78 (131.33, 277.43)	−0.14 (−21.64, 27.86)	0.25 (0.09, 0.41)
Australasia	1,826.72 (1,712.05, 1,933.55)	46.37 (43.46, 49.08)	1,492.29 (1,298.04, 1,620.05)	16.89 (14.69, 18.34)	−18.31 (−26.43, −11.80)	−63.57 (−67.19, −60.67)	−3.91 (−4.12, −3.70)
Caribbean	855.56 (790.97, 913.64)	19.85 (18.35, 21.20)	1,318.58 (1,159.34, 1,476.95)	14.24 (12.52, 15.95)	54.12 (35.95, 73.16)	−28.26 (−36.72, −19.40)	−1.43 (−1.60, −1.26)
Central Asia	336.45 (289.44, 402.37)	4.21 (3.62, 5.03)	1,234.08 (1,095.70, 1,383.65)	8.48 (7.53, 9.51)	266.79 (198.04, 339.71)	101.63 (63.84, 141.71)	2.46 (2.28, 2.65)
Central Europe	3,847.21 (3,687.46, 3,977.96)	14.51 (13.90, 15.00)	6,213.49 (5,716.40, 6,801.20)	16.78 (15.44, 18.37)	61.51 (49.56, 76.63)	15.67 (7.12, 26.51)	0.12 (−0.07, 0.31)
Central Latin America	987.94 (942.54, 1,027.58)	7.28 (6.95, 7.57)	2,908.56 (2,525.26, 3,328.30)	6.80 (5.90, 7.78)	194.41 (157.08, 236.30)	−6.58 (−18.43, 6.71)	−1.01 (−1.32, −0.71)
Central Sub-Saharan Africa	400.39 (217.61, 667.45)	10.65 (5.79, 17.75)	856.67 (477.70, 1,397.99)	9.49 (5.29, 15.49)	113.96 (61.29, 183.37)	−10.84 (−32.79, 18.08)	−0.59 (−0.75, −0.44)
East Asia	1,849.72 (1,512.66, 2,299.32)	1.24 (1.02, 1.54)	7,699.48 (6,220.92, 9,524.14)	1.96 (1.59, 2.43)	316.25 (192.52, 474.42)	58.12 (11.12, 118.20)	1.54 (1.42, 1.66)
Eastern Europe	5,640.40 (5,427.65, 5,884.70)	11.54 (11.10, 12.04)	11,842.32 (10,896.40, 12,763.24)	19.08 (17.55, 20.56)	109.96 (92.54, 126.98)	65.36 (51.65, 78.77)	1.31 (0.99, 1.63)
Eastern Sub-Saharan Africa	911.76 (533.71, 1,555.47)	7.49 (4.39, 12.79)	2,037.54 (1,104.09, 3,371.79)	7.54 (4.08, 12.47)	123.47 (66.78, 200.84)	0.55 (−24.96, 35.37)	−0.21 (−0.37, −0.05)
High-income Asia Pacific	4,894.03 (4,516.10, 5,125.51)	14.00 (12.91, 14.66)	24,857.23 (20,020.26, 27,617.45)	35.26 (28.40, 39.17)	407.91 (341.50, 454.19)	151.91 (118.98, 174.87)	3.18 (3.05, 3.31)
High-income North America	18,539.85 (17,178.63, 19,326.41)	32.00 (29.66, 33.36)	12,703.78 (11,188.78, 13,505.33)	11.29 (9.94, 12.00)	−31.48 (−34.98, −29.10)	−64.73 (−66.53, −63.51)	−4.22 (−4.55, −3.89)
North Africa and Middle East	766.43 (573.16, 1,035.34)	2.71 (2.03, 3.66)	2,932.09 (2,522.52, 3,433.24)	3.85 (3.31, 4.50)	282.56 (166.76, 439.21)	41.84 (−1.10, 99.91)	1.33 (1.21, 1.45)
Oceania	37.26 (28.44, 49.94)	7.75 (5.91, 10.38)	87.15 (67.88, 110.86)	7.06 (5.50, 8.98)	133.86 (88.29, 194.57)	−8.84 (−26.60, 14.83)	−0.53 (−0.65, −0.42)
South Asia	2,840.08 (1,796.93, 4,566.73)	2.99 (1.89, 4.81)	13,965.31 (10,035.92, 20,377.61)	5.62 (4.04, 8.21)	391.72 (267.06, 581.29)	88.02 (40.35, 160.51)	2.17 (2.07, 2.27)
Southeast Asia	1,738.50 (1,397.58, 2,180.37)	4.11 (3.30, 5.15)	6,540.62 (5,719.64, 7,554.84)	5.71 (4.99, 6.59)	276.22 (194.00, 376.33)	39.06 (8.67, 76.06)	0.90 (0.73, 1.07)
Southern Latin America	1,914.56 (1,777.46, 2,074.10)	24.17 (22.44, 26.18)	2,149.33 (1,971.42, 2,315.96)	14.61 (13.40, 15.74)	12.26 (−0.14, 25.04)	−39.57 (−46.25, −32.69)	−1.84 (−2.12, −1.56)
Southern Sub-Saharan Africa	586.50 (460.12, 706.69)	13.25 (10.40, 15.97)	995.40 (902.88, 1,093.07)	10.22 (9.27, 11.23)	69.72 (41.49, 121.68)	−22.86 (−35.69, 0.76)	−1.50 (−1.89, −1.10)
Tropical Latin America	2,245.90 (2,134.43, 2,334.05)	14.83 (14.10, 15.42)	8,764.08 (7,980.25, 9,279.91)	19.78 (18.01, 20.95)	290.23 (265.49, 310.71)	33.38 (24.93, 40.38)	0.65 (0.34, 0.96)
Western Europe	27,754.95 (26,032.69, 28,621.87)	28.58 (26.81, 29.47)	26,510.00 (23,076.72, 28,162.70)	17.78 (15.47, 18.88)	−4.49 (−11.39, −0.28)	−37.80 (−42.30, −35.07)	−1.99 (−2.24, −1.74)
Western Sub-Saharan Africa	1,477.30 (780.91, 2,627.39)	10.23 (5.41, 18.20)	2,882.18 (1,444.99, 4,848.08)	8.97 (4.50, 15.08)	95.10 (45.92, 144.01)	−12.38 (−34.47, 9.59)	−0.71 (−0.85, −0.56)

EAPC, estimated annual percentage change; SDI, Sociodemographic Index; UI, uncertainty interval.

^a^ Change shows the percentage change.

^b^ EAPC is expressed as 95% confidence interval.

#### DALYs

Consistent with mortality trends, global AA-related DALY rates initially rose before declining over the last three decades. The highest APC (1.09%; 95% CI, 0.76%–1.42%) was recorded between 1990 and 1994, peaking at 229.32 per 100,000 in 1994 (95% UI, 217.00–242.16; Figure 1D). The peak for males occurred simultaneously, with an APC of 0.92% (95% CI, 0.61%–1.24%) and DALY rate of 331.39 per 100,000 (95% UI, 312.88–357.98; Figure 1E), while females peaked in 1999 (142.06 per 100,000; 95% UI, 128.81–155.97) with an APC of 1.29% (95% CI, 0.84%–1.75%; Figure 1F). Global DALYs rose by 62.57%, from 1,475,228.48 (95% UI, 1,399,236.58–1,556,633.48) in 1990 to 2,398,319.66 (95% UI, 2,194,896.37–2,576,073.74) in 2021. DALY rates decreased from 219.72 per 100,000 in 1990 to 161.40 per 100,000 in 2021, a decline of 26.54% (95% UI, −31.02% to −22.76%), with an EAPC of −1.33 (95% CI, −1.47 to −1.20; Table 2). Male DALY rates consistently exceeded those of females across all age groups, with the highest DALY rates occurring among individuals over 95, accounting for 25.7% of all DALYs at 733.79 per 100,000 (95% UI, 514.20–852.90; Figure 3B).

**Table 2:** DALYs of aortic aneurysm between 1990 and 2021 at the global and regional level.

Location	1990 (95% UI)		2021 (95% UI)		1990–2021 (95% UI)		
	DALY cases	DALY rate	DALY cases	DALY rate	Cases change^a^	Rate change^a^	EAPC^b^
Global	1,475,228.48 (1,399,236.58, 1,556,633.48)	219.72 (208.40, 231.84)	2,398,319.66 (2,194,896.37, 2,576,073.74)	161.40 (147.71, 173.36)	62.57 (52.66, 70.94)	−26.54 (−31.02, −22.76)	−1.33 (−1.47, −1.20)
High SDI	896,446.67 (851,955.63, 919,213.45)	480.76 (456.90, 492.97)	963,979.62 (852,859.40, 1,023,695.99)	279.40 (247.20, 296.71)	7.53 (0.06, 12.52)	−41.88 (−45.92, −39.19)	−2.19 (−2.35, −2.03)
High-middle SDI	314,639.73 (302,310.16, 328,014.51)	182.37 (175.23, 190.13)	578,164.60 (540,852.76, 618,522.80)	166.77 (156.01, 178.41)	83.75 (68.56, 98.06)	−8.55 (−16.12, −1.43)	−0.69 (−0.88, −0.49)
Middle SDI	139,043.21 (128,624.04, 155,299.59)	80.11 (74.11, 89.48)	466,076.23 (423,566.32, 505,897.54)	99.20 (90.15, 107.67)	235.20 (193.33, 274.52)	23.82 (8.35, 38.34)	0.39 (0.21, 0.57)
Low-middle SDI	78,036.31 (61,579.97, 107,305.01)	77.42 (61.09, 106.45)	281,353.94 (234,208.16, 376,926.65)	116.70 (97.15, 156.35)	260.54 (197.67, 329.37)	50.75 (24.46, 79.53)	1.29 (1.23, 1.35)
Low SDI	44,779.99 (27,161.54, 77,917.68)	120.03 (72.80, 208.85)	105,606.19 (65,459.56, 174,699.24)	128.70 (79.77, 212.90)	135.83 (92.79, 189.80)	7.22 (−12.35, 31.76)	0.09 (−0.09, 0.28)
Regions							
Andean Latin America	2,914.00 (2,438.22, 3,502.07)	86.83 (72.66, 104.36)	8,342.91 (6,920.79, 10,100.37)	84.22 (69.86, 101.96)	186.30 (120.34, 273.69)	−3.01 (−25.36, 26.59)	0.04 (−0.10, 0.18)
Australasia	32,079.18 (30,288.80, 33,918.66)	814.29 (768.85, 860.99)	22,412.10 (19,983.56, 24,075.12)	253.70 (226.21, 272.52)	−30.14 (−36.26, −24.87)	−68.84 (−71.58, −66.49)	−4.43 (−4.64, −4.22)
Caribbean	15,160.08 (14,106.14, 16,243.45)	351.76 (327.31, 376.90)	22,943.78 (20,226.07, 25,951.81)	247.81 (218.46, 280.30)	51.34 (33.26, 70.72)	−29.55 (−37.97, −20.53)	−1.47 (−1.63, −1.30)
Central Asia	7,345.66 (6,378.44, 8,705.22)	91.84 (79.75, 108.84)	25,639.18 (22,574.10, 28,910.49)	176.22 (155.15, 198.70)	249.04 (185.53, 317.09)	91.87 (56.96, 129.27)	2.09 (1.92, 2.26)
Central Europe	77,643.60 (74,984.66, 80,121.88)	292.77 (282.74, 302.11)	113,914.44 (104,729.96, 125,138.91)	307.64 (282.84, 337.96)	46.71 (34.40, 62.12)	5.08 (−3.74, 16.11)	−0.24 (−0.43, −0.05)
Central Latin America	19,635.58 (18,831.87, 20,400.02)	144.70 (138.77, 150.33)	54,006.75 (46,622.31, 62,315.04)	126.28 (109.02, 145.71)	175.05 (137.51, 217.52)	−12.73 (−24.63, 0.75)	−1.32 (−1.64, −1.01)
Central Sub-Saharan Africa	8,914.94 (4,850.56, 15,021.54)	237.08 (128.99, 399.48)	18,662.94 (10,325.75, 30,409.13)	206.83 (114.43, 337.00)	109.34 (54.95, 179.82)	−12.76 (−35.43, 16.61)	−0.67 (−0.87, −0.48)
East Asia	40,900.13 (33,094.11, 51,932.57)	27.46 (22.22, 34.87)	154,301.09 (123,710.31, 193,835.77)	39.35 (31.55, 49.43)	277.26 (158.18, 433.77)	43.31 (−1.93, 102.76)	1.24 (1.14, 1.35)
Eastern Europe	119,888.14 (115,772.23, 124,923.85)	245.21 (236.79, 255.51)	235,419.75 (216,564.39, 254,630.43)	379.23 (348.85, 410.17)	96.37 (79.88, 112.88)	54.66 (41.67, 67.66)	1.00 (0.71, 1.29)
Eastern Sub-Saharan Africa	19,124.18 (11,094.15, 32,899.55)	157.20 (91.19, 270.43)	41,812.74 (22,376.41, 68,779.90)	154.65 (82.76, 254.39)	118.64 (62.31, 197.91)	−1.62 (−26.97, 34.05)	−0.30 (−0.47, −0.14)
High-income Asia Pacific	87,665.06 (82,105.39, 91,824.19)	250.70 (234.80, 262.59)	339,215.31 (286,436.34, 369,497.87)	481.13 (406.27, 524.09)	286.94 (246.83, 317.11)	91.92 (72.02, 106.88)	2.26 (2.13, 2.39)
High-income North America	327,225.66 (309,176.81, 337,899.72)	564.88 (533.73, 583.31)	214,102.20 (195,517.01, 224,570.68)	190.26 (173.74, 199.56)	−34.57 (−37.00, −32.56)	−66.32 (−67.57, −65.28)	−4.36 (−4.69, −4.04)
North Africa and Middle East	17,119.21 (12,621.83, 23,873.77)	60.57 (44.66, 84.47)	60,349.87 (51,738.89, 70,830.77)	79.16 (67.87, 92.91)	252.53 (137.26, 409.63)	30.70 (−12.03, 88.94)	0.94 (0.82, 1.06)
Oceania	822.48 (614.98, 1,138.19)	170.96 (127.83, 236.59)	1,877.49 (1,434.82, 2,441.74)	152.13 (116.26, 197.85)	128.27 (79.54, 194.06)	−11.02 (−30.01, 14.63)	−0.59 (−0.68, −0.49)
South Asia	60,281.25 (37,935.92, 98,457.20)	63.49 (39.96, 103.70)	274,322.76 (196,039.35, 401,991.22)	110.48 (78.95, 161.90)	355.07 (240.42, 535.43)	74.01 (30.17, 142.97)	1.83 (1.73, 1.93)
Southeast Asia	32,299.73 (26,056.29, 40,521.55)	76.28 (61.54, 95.70)	117,485.80 (102,456.19, 134,677.11)	102.56 (89.44, 117.57)	263.74 (181.41, 363.00)	34.44 (4.01, 71.13)	0.78 (0.63, 0.92)
Southern Latin America	37,687.28 (34,979.92, 41,141.94)	475.76 (441.58, 519.37)	39,567.15 (36,497.40, 42,739.69)	268.87 (248.01, 290.43)	4.99 (−7.28, 18.09)	−43.49 (−50.09, −36.44)	−2.08 (−2.34, −1.82)
Southern Sub-Saharan Africa	10,710.63 (8,448.95, 12,849.52)	242.06 (190.95, 290.40)	19,421.10 (17,564.71, 21,325.56)	199.49 (180.42, 219.05)	81.33 (51.63, 135.60)	−17.59 (−31.08, 7.08)	−1.26 (−1.66, −0.87)
Tropical Latin America	48,259.06 (46,068.07, 49,987.60)	318.72 (304.25, 330.14)	171,843.15 (158,938.06, 181,235.74)	387.93 (358.79, 409.13)	256.08 (234.40, 274.31)	21.71 (14.30, 27.94)	0.29 (−0.02, 0.60)
Western Europe	480,708.18 (458,128.22, 493,057.60)	495.00 (471.75, 507.72)	406,059.17 (365,383.46, 427,589.00)	272.28 (245.00, 286.71)	−15.53 (−20.45, −12.08)	−44.99 (−48.20, −42.75)	−2.43 (−2.68, −2.18)
Western Sub-Saharan Africa	28,844.46 (15,090.62, 51,809.23)	199.82 (104.54, 358.90)	56,620.00 (28,133.95, 96,164.48)	176.15 (87.53, 299.18)	96.29 (45.40, 148.69)	−11.84 (−34.70, 11.69)	−0.72 (−0.87, −0.57)

EAPC, estimated annual percentage change; SDI, sociodemographic Index; UI, uncertainty interval.

^a^ Change shows the percentage change.

^b^ EAPC is expressed as 95% confidence interval.

### AA in adults over 55: SDI regional trends

Compared to 1990, all five SDI regions experienced an increase in AA-related mortality by 2021, with the Low-middle SDI region showing the greatest increase (277.51%; 95% UI, 213.93–345.28%), corresponding to a mortality rate increase from 3.76 to 5.94 per 100,000 [57.84% increase; EAPC, 1.49 (95% CI, 1.43–1.54); Table 1]. Similarly, AA-related DALYs rose in all SDI regions, with Low-middle SDI experiencing the largest growth (260.54%; 95% UI, 197.67–329.37%) and an increase in DALY rate from 77.42 to 116.70 per 100,000 [50.75% increase; EAPC, 1.29 (95% CI, 1.23–1.35); Table 2].

### AA in adults over 55: geographic regional trends

#### Mortality

**Figure 4:**
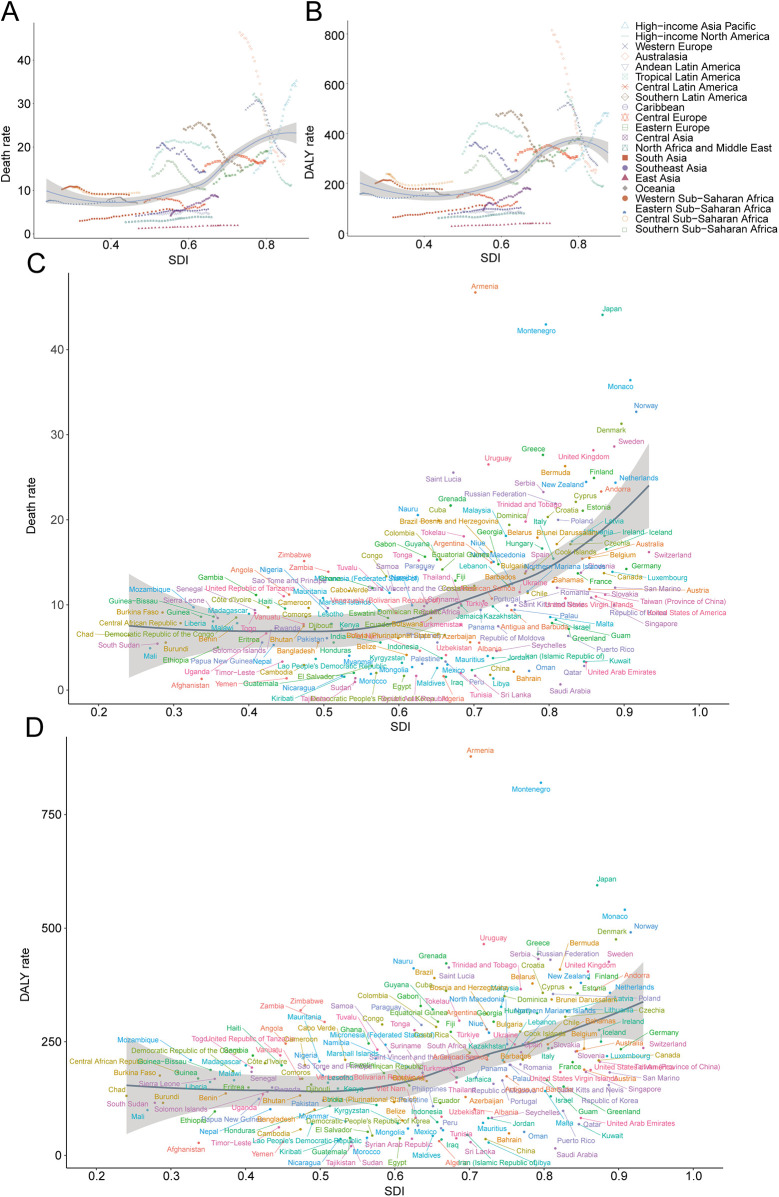
Trends of death and disability-adjusted life-years (DALYs) rates in regions of aortic aneurysm from 1990 to 2021. **(A)** Death rate (regional trends); **(B)** DALY rate (regional trends); **(C)** death rate (national trends); **(D)** DALY rate (national trends).

In 2021, Western Europe reported the highest absolute number of AA-related deaths (26,510.0; 95% UI, 23,076.7–28,162.7), whereas the High-income Asia Pacific region recorded the highest mortality rate (35.26 per 100,000; 95% UI, 28.40–39.17). The most significant decrease in AA-related mortality rates was observed in High-income North America, with an estimated annual percentage change (EAPC) of −4.22 (95% CI, −4.55 to −3.89). Conversely, High-income Asia Pacific exhibited the largest increase (EAPC, 3.18; 95% CI, 3.05–3.31). Globally, with an average SDI of 0.67, AA mortality rates exceeded the global mean (9.32 per 100,000) in 11 regions, whereas 10 regions were below this global average (Table 1, Figure 4A).

#### DALYs

Western Europe accounted for the highest absolute number of AA-related DALYs in 2021 (406,059.2; 95% UI, 365,383.5–427,589.0). However, High-income Asia Pacific showed the highest DALY rate (481.13 per 100,000; 95% UI, 406.27–524.09). Australasia recorded the greatest reduction in DALY rates from 1990 to 2021 (EAPC, −4.43; 95% CI, −4.64 to −4.22), while High-income Asia Pacific demonstrated the most notable increase (EAPC, 2.26; 95% CI, 2.13–2.39). Thirteen regions, including High-income Asia Pacific, surpassed the global DALY rate average (161.40 per 100,000), whereas eight regions, such as North Africa and the Middle East, fell below this level (Table 2, Figure 4B).

### AA in adults over 55: national trends

#### Mortality

**Figure 5:**
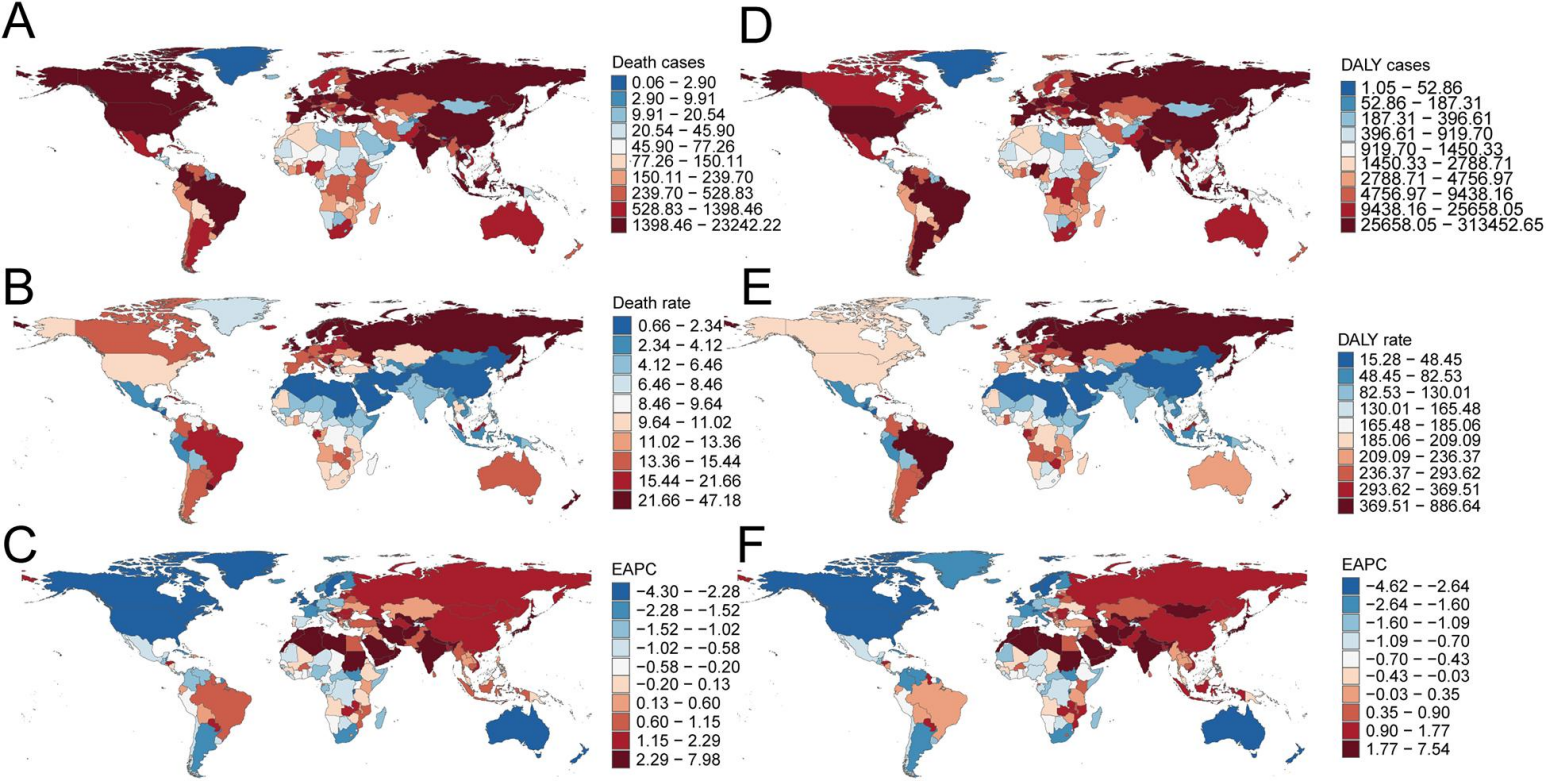
Death and disability-adjusted life-years (DALYs) cases of aortic aneurysm in 204 countries and territories. **(A)** Death cases; **(B)** death rate; **(C)** EAPC for death rate; **(D)** DALY cases; **(E)** DALY rate; **(F)** EAPC for DALY rate.

Japan registered the highest total AA-related deaths in 2021 (23,012.1; 95% UI, 18,379.5–25,666.3), whereas Armenia exhibited the highest national AA mortality rate (46.72 per 100,000; 95% UI, 38.80–54.98). The steepest increase in AA-related mortality was observed in Georgia (EAPC, 7.90; 95% CI, 6.61–9.19), with America experiencing the greatest reduction (EAPC, −4.26; 95% CI, −4.59 to −3.92). Worldwide, the AA mortality rate averaged 9.32 per 100,000 (95% UI, 8.33–10.04), with 110 countries exceeding this average and 94 countries below (Table 1, Supplementary Table S1, Figures 4C, 5A–C).

#### DALYs

In 2021, Japan had the highest number of AA-related DALYs (310,349.2; 95% UI, 260,095.4–337,781.0), whereas Armenia recorded the highest DALY rate (877.86 per 100,000; 95% UI, 729.13–1,035.52). Australia exhibited the largest reduction in DALY rates (EAPC, −4.57; 95% CI, −4.78 to −4.35), while Georgia showed the greatest increase (EAPC, 7.47; 95% CI, 6.15–8.82). Globally, AA DALY rates were 161.40 per 100,000 (95% UI, 147.71–173.36), with 127 countries exceeding and 77 countries below this global benchmark (Table 2, Supplementary Table S2, Figures 4D, 5D–F).

### Risk factors for AA in adults over 55

**Figure 6:**
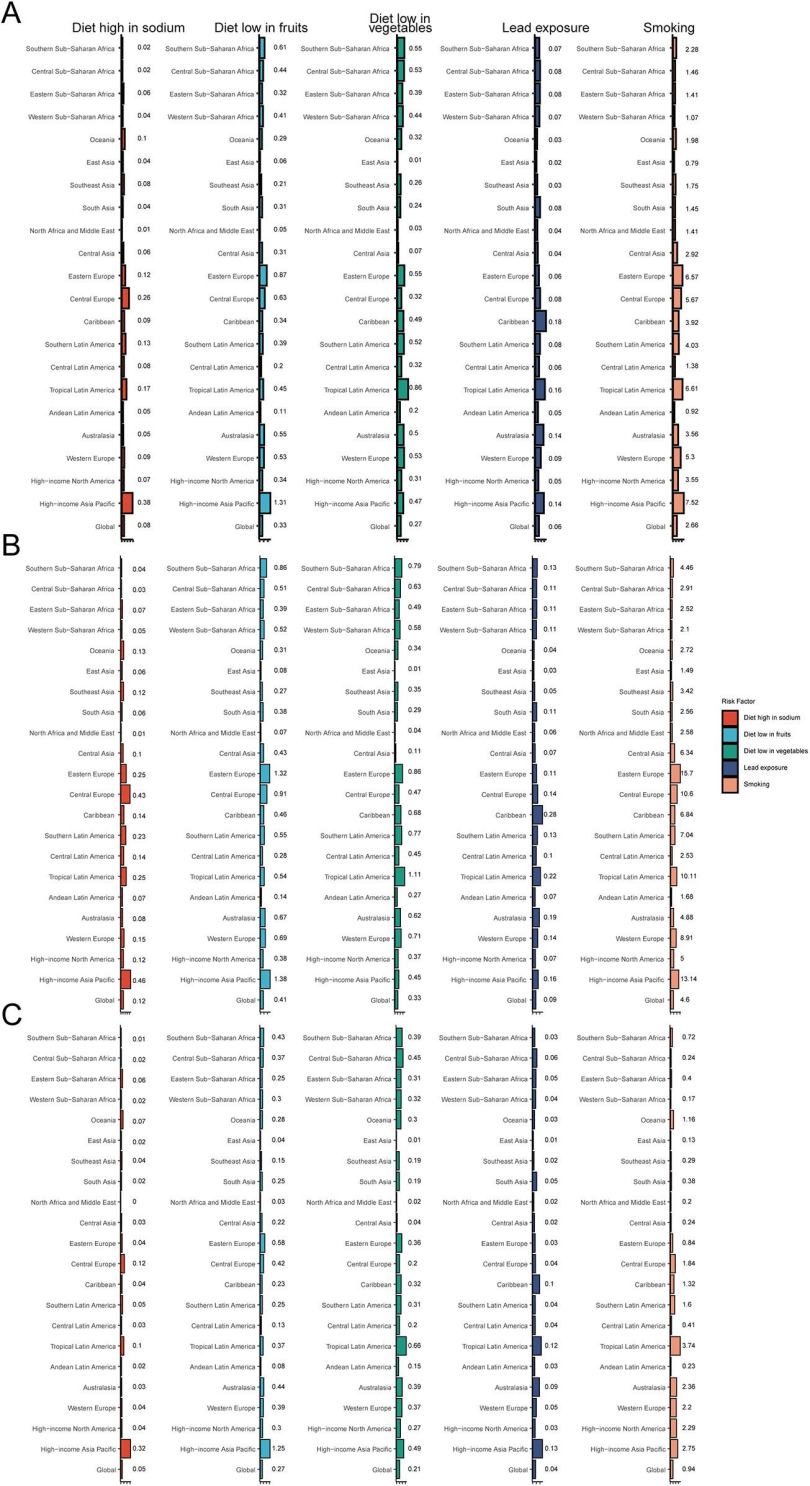
Aortic aneurysm deaths attributable to key risk factors. **(A)** Both sexes; **(B)** males; **(C)** females.

In 2021, the primary global risk factors associated with AA included diets high in sodium, diets low in fruits and vegetables, lead exposure, and smoking, with smoking identified as the predominant risk factor, accounting for a mortality rate of 2.66 per 100,000 (95% UI, 2.17–3.19). Male mortality due to smoking (4.60 per 100,000; 95% UI, 3.81–5.43) was substantially higher than female mortality (0.94 per 100,000; 95% UI, 0.71–1.22) (Figures 6A–C).

### Future trend for AA burden from 2022 to 2035

**Figure 7:**
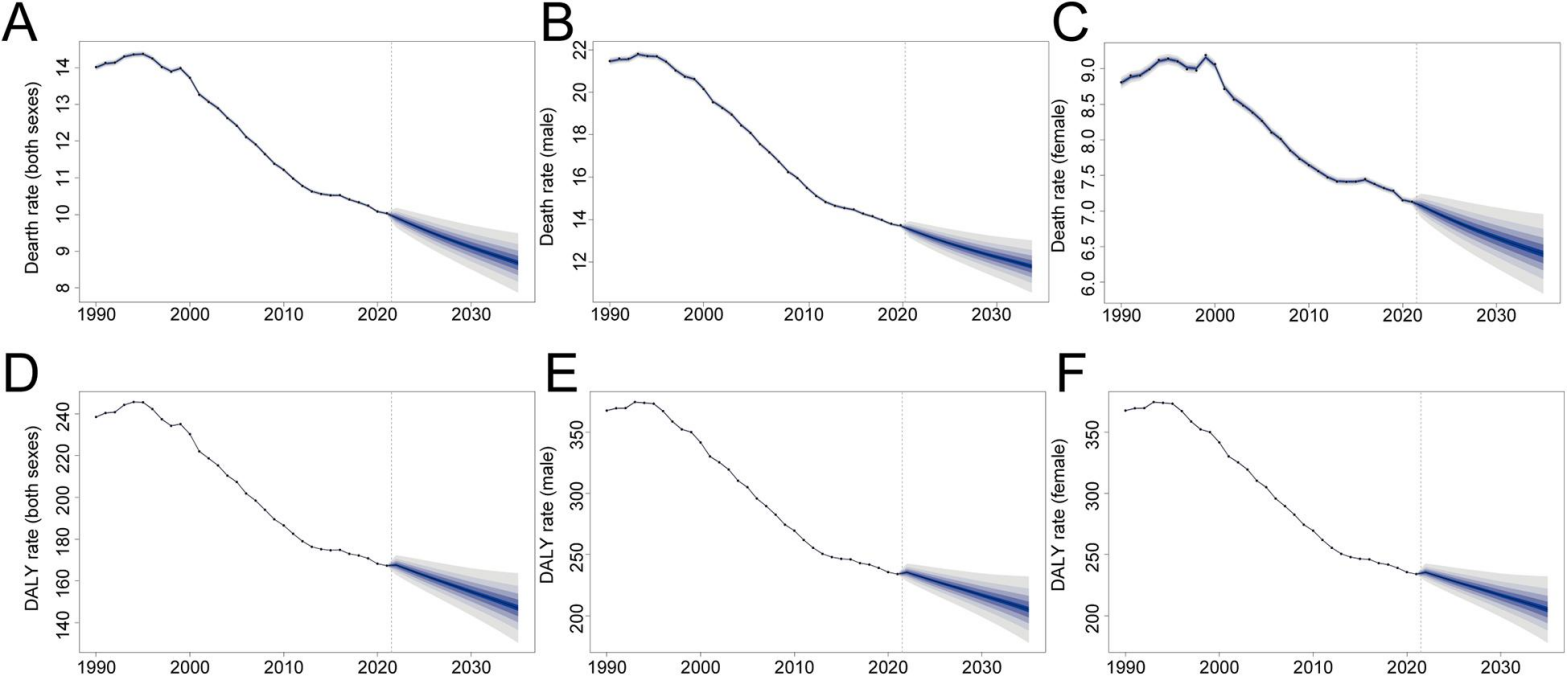
The change trends of the aortic aneurysm burden by sex from 1990 to 2035. **(A)** Death rate (both sexes); **(B)** death rate (males); **(C)** death rate (females); **(D)** disability-adjusted life-year (DALY) rate (both sexes); **(E)** DALY rate (males); **(F)** DALY rate (females).

Based on BAPC modeling, the AA burden is expected to decline gradually by 2035. Projected global mortality rates are estimated at 8.68 per 100,000 overall, 11.79 per 100,000 for males, and 6.40 per 100,000 for females. DALY rates are similarly expected to decrease, with projected values of 147.06 per 100,000 overall, 204.98 per 100,000 for males, and 100.99 per 100,000 for females (Figures 7A–F).

## Discussion

In this study, we present the first systematic epidemiological evaluation using data from the GBD study spanning 1990 to 2021. This study delineates global mortality and DALYs, stratified by region, country, gender, and age group. The findings furnish essential, high-quality evidence to inform public health policy and enable the development of precisely targeted prevention and intervention strategies.

Consistent with known epidemiology, men bear a disproportionately higher burden of AA than women (23, 24). We found that males not only have significantly higher age-specific death rates but also saw larger absolute increases in aneurysm deaths over time, likely because more men historically were exposed to risk factors like smoking. However, the gap has shown signs of narrowing: the decline in age-adjusted aneurysm mortality has been steeper in men than in women in recent decades (e.g., a 24% decline in men vs. only ∼9% in women from 1990 to 2019) (17). This may reflect that male rates were higher to begin with (allowing more room for improvement) and that female smokers of past generations have been catching up in risk. In terms of age, our results reaffirm that AA is predominantly a disease of older adults. Individuals over 70 years of age accounted for most aneurysm deaths globally. Notably, males tend to die from AA at somewhat younger ages than females – in 2021, the peak mortality in men occurred around 70–74 years, whereas in women it was at 80–84 years. This is explained by both the later onset of disease in women and their longer life expectancy (women who survive into very old age may outnumber men and thus comprise more very-late-age aneurysm deaths).

Changing patterns of risk factor exposure are another critical determinant of aneurysm trends. Chief among these is smoking. Cigarette smoking has a causal, cumulative effect on AA risk, and historical smoking patterns often predict current aneurysm outcomes (25). Many high-income countries saw peak smoking prevalence in the mid-20th century, followed by substantial declines in recent decades (26); correspondingly, their AA mortality rates are now falling. By contrast, regions where smoking rates remain high or declined later (such as Eastern Europe, parts of Asia, and some developing countries with rising tobacco use) have not seen similar improvements (27). Our analysis of risk-attributable burden confirms that smoking remains the leading modifiable contributor to aneurysm mortality, especially for men. Globally, an estimated one-third of all AA deaths in males are attributable to smoking, and in high-burden areas, the fraction is even higher. In Eastern European males, over 60% of AAA mortality was linked to smoking, a testament to the decades of heavy tobacco use in that region. In women, who historically smoked less, high blood pressure appears to play a relatively larger role (population-attributable fraction ∼34% for elevated systolic blood pressure in females vs. ∼33% for smoking in males) (17). Encouragingly, many of these risk factors have been improving in high-SDI countries; smoking prevalence, mean blood pressure levels, and other contributors like high cholesterol have all shown declining trends, especially in Western nations. This risk factor decline has likely translated into fewer new aneurysms and slower expansion of existing ones, thereby reducing rupture incidence. In lower-SDI regions, however, risk factor trends are a mixed picture: some have seen increases in tobacco use (as the tobacco epidemic shifts to new markets) or persistently high hypertension prevalence due to limited healthcare access. These unfavorable risk profiles can explain why aneurysm rates have stagnated or risen in certain populations despite global medical advances.

Apart from risk factor prevention, improvements in medical care and diagnostics have influenced aneurysm outcomes. The introduction of widespread ultrasound screening for AAA in several high-income countries (such as the UK, parts of Europe, and the US for selected groups) in the 2000s enabled the detection of asymptomatic aneurysms and prophylactic surgical repair for large aneurysms (28). This has been credited with significant reductions in aneurysm rupture and death in those screened populations. Likewise, advances in surgical techniques, notably the development of endovascular aneurysm repair (EVAR) in the 1990s, have made elective treatment of AAA safer and more accessible, contributing to improved survival (29). Regions that implemented these innovations early (Australasia, North America, Western Europe) saw notable mortality declines. In contrast, many low- and middle-income countries lack routine screening programs and have limited capacity for elective aneurysm surgery, meaning aneurysms often go undetected until rupture. The availability of emergency care for ruptured aneurysms also varies: timely surgical intervention for rupture can be life-saving, but requires robust emergency transport and surgical infrastructure, which may be lacking in rural or resource-poor areas. Additionally, increased utilization of diagnostic imaging (ultrasound, CT, MRI) in high-income settings has likely improved the accuracy of cause-of-death attribution for aneurysms (fewer ruptures being misclassified as other causes), which might inflate reported aneurysm mortality initially, even as true incidence falls (30). Improved diagnostics can also identify more aneurysms incidentally, potentially increasing recorded prevalence, although this would reduce mortality if treated. Thus, differences in health system capacity and practices, including preventive screening, elective surgery availability, and acute care, are key to understanding the divergent aneurysm trends. Regions that combined risk factor reduction with proactive aneurysm detection and treatment have reaped the largest gains, whereas those lagging in these interventions face a mounting burden.

Our findings carry important implications for global health policy and clinical practice regarding aortic aneurysms. Given the strong influence of modifiable risks and the severe consequences of aneurysm rupture, a multifaceted strategy is warranted to curb the growing burden:

• **Strengthen tobacco control:** Reducing smoking prevalence is the single most effective measure to prevent future aneurysm cases. The high fraction of AAA deaths attributable to smoking, especially in men, underscores the need for sustained tobacco control policies worldwide. This includes higher tobacco taxes, public smoking bans, anti-tobacco media campaigns, and smoking cessation support programs. Countries in Eastern Europe, Asia, and other regions with rising or high smoking rates should be prioritized for aggressive tobacco control to alter the trajectory of AAA burden in the coming decades.
• **Manage hypertension and cardiovascular risks:** Integrated cardiovascular risk reduction will also pay dividends in lowering aneurysm mortality. Improved hypertension control (through increased screening and access to antihypertensive therapy) can slow aneurysm expansion and reduce rupture risk, particularly in women. Likewise, promoting healthy diets (low in sodium, balanced in lipids) and treating hyperlipidemia could have ancillary benefits for aneurysm prevention, given the shared risk with atherosclerosis. These interventions should be part of primary care strengthening in low- and middle-income countries, as they combat multiple non-communicable diseases alongside aneurysms.
• **Implement targeted screening programs:** Where resources allow, screening for abdominal aortic aneurysm in high-risk groups can substantially reduce mortality by enabling early intervention. Randomized trials in high-income countries have shown that ultrasound screening of men over 65 who have ever smoked is cost-effective and reduces AAA rupture and death. The success of screening in developed nations suggests that similar programs could be adapted in emerging economies with growing elderly populations and high smoking prevalence. Screening efforts might initially focus on subsets of the population (e.g., men ≥65 with smoking history, or individuals with a family history of aneurysm), especially in regions now experiencing increased AAA incidence (such as parts of the Asia-Pacific). Any screening program should be coupled with the capacity to offer elective surgical repair (open or endovascular) for those aneurysms that meet size/intervention criteria.
• **Enhance surgical and emergency care capacity:** Health systems, particularly in low-SDI settings, should be bolstered to handle aortic aneurysm treatment. This includes training vascular surgeons and interventional radiologists, expanding facilities for endovascular aneurysm repair, and improving emergency response systems for acute aortic catastrophes. While prevention is paramount, not all aneurysms will be prevented; having the infrastructure to treat large aneurysms electively and to manage ruptures (through rapid transport and on-call surgical teams) can significantly improve survival outcomes. International collaborations and funding could help bring life-saving surgical capabilities to regions currently lacking them.
• **Surveillance and research:** As AA epidemiology evolves, continued surveillance is necessary. Countries should invest in robust vital registration and cause-of-death reporting systems to accurately monitor AA mortality. Additionally, further research is needed to identify why certain populations (e.g., some high-income Asian countries or certain Latin American nations) have higher AA risk and to develop tailored interventions. Genetic and biochemical research into aneurysm pathogenesis could also yield biomarkers for earlier detection or pharmaceutical therapies to slow AA growth.

### Clinical implications for care

#### Risk-targeted AAA screening

Offer one-time abdominal ultrasound to men ≥65 years with any smoking history (and consider men ≥65 without smoking and women ≥65 with strong family history, where resources allow) to enable pre-rupture detection and elective repair.

#### Aggressive tobacco cessation

Embed opt-out cessation (brief advice, pharmacotherapy, and behavioral support) in vascular clinics and primary care for patients with known aneurysms or at high risk.

#### Tight blood pressure control

Prioritize SBP control, especially in women and patients with thoracic aortic disease, to slow expansion and reduce rupture risk; ensure rapid treatment of hypertensive crises.

#### Cardiovascular risk bundling

Manage lipids, diabetes, and smoking to reduce **overall cardiovascular events** in aneurysm patients; while this may not directly modify aneurysm biology, it improves survival and perioperative outcomes.

Overall, AA needs to be recognized as an important component of the global non-communicable disease (NCD) agenda. Many of the tools to reduce its burden – tobacco control, blood pressure management, and ultrasound screening – are available and align with general NCD prevention strategies. The challenge lies in implementing these measures widely, including in lower-resource settings, before AA rates escalate further.

## Limitations

This study has several limitations that merit consideration. First, our estimates rely on the GBD framework, which synthesizes heterogeneous data sources using standardized statistical models. As a result, the quality, completeness, and comparability of our findings are constrained by the quality of the input data. Civil registration and vital statistics coverage, diagnostic and imaging capacity, coding practices, and survey representativeness vary widely across locations and over time. Although GBD applies bias-correction and cause-of-death redistribution, residual error is inevitable and may differentially affect low- and middle-SDI settings. Second, cause-of-death misclassification likely leads to underestimation of aneurysm mortality. Experience from emergency departments that routinely employ post-mortem CT suggests that a substantial fraction of aneurysm-related deaths are certified as “myocardial infarction”, thereby escaping aneurysm codes. While redistribution algorithms attempt to reallocate ill-defined or competing causes, systematic misclassification of this kind is difficult to fully correct and may bias both absolute levels and temporal trends. Third, access to diagnostic imaging is uneven across health systems and may produce ascertainment bias. Limited availability of ultrasound or cross-sectional imaging can shift the clinical spectrum from screen-detected disease to undetected rupture, inflating observed mortality without reflecting true incidence. Conversely, expansion of screening and surveillance programs can increase case detection and elective repair rates, altering recorded incidence and case-fatality independently of underlying biology. Finally, if projections are considered, they should be viewed as conditional forecasts subject to modeling assumptions about demographic change, risk factor trajectories, and future healthcare access. Structural breaks (e.g., screening policy shifts, technology adoption, or coding reclassifications) could lead to deviations from projected paths. Together, these limitations do not negate the broad patterns we report, but they do warrant cautious interpretation of absolute levels, geographic micro-gradients, and mechanistic inferences.

## Conclusion

In summary, the global burden of AA has increased substantially over the past three decades in terms of deaths and disability, despite significant declines in mortality and DALY rates. This paradox reflects the profound effects of demographic change and persistent exposure to modifiable risk factors, particularly in low- and middle-SDI regions. Targeted prevention strategies—chiefly tobacco control, hypertension management, and high-risk group screening—combined with enhanced surgical capacity, are urgently needed to address the rising absolute burden and close the gap between regions. Ongoing surveillance and research will be vital to inform public health action and realize further reductions in AA-related mortality worldwide.

## Data Availability

The original contributions presented in the study are included in the article/Supplementary Material, further inquiries can be directed to the corresponding author.
