# Supplementary material for: The global burden of aortic aneurysm in adults over 55: evolving trends, risk factors, and projections
Source: Front Cardiovasc Med. 2025 Nov 3;12:1629635. doi: 10.3389/fcvm.2025.1629635 (PMC12620971; doi:10.3389/fcvm.2025.1629635)
Supplement: Supplementary file 1 [file Table1.pdf]

Table S1. Mortality of Aortic Aneurysm between 1990 and 2021 at national level.

location	1990		2021		1990-2021		
	Death cases	Death rate	Death cases	Death rate	Cases change	Rate change	EAPC
Afghanistan	4.34(2.30,7.52)	0.36(0.19,0.62)	16.06(10.27,22.20)	1.31(0.84,1.81)	270.06(153.40,470.46)	265.97(150.60,464.16)	5.35(4.99,5.72)
Albania	14.49(11.89,17.18)	4.17(3.42,4.95)	43.67(30.80,59.84)	5.55(3.92,7.61)	201.48(100.30,345.70)	33.06(-11.60,96.71)	1.19(1.03,1.35)
Algeria	11.92(8.39,16.37)	0.57(0.40,0.78)	98.46(73.09,130.06)	1.62(1.20,2.14)	726.23(462.58,1100.94)	185.01(94.06,314.27)	4.15(3.77,4.53)
American Samoa	0.50(0.42,0.59)	13.78(11.61,16.13)	0.91(0.75,1.10)	10.74(8.88,13.04)	80.03(41.63,130.47)	-22.05(-38.68,-0.22)	-1.12(-1.32,-0.92)
Andorra	3.43(2.17,5.15)	34.78(22.03,52.16)	6.17(3.95,9.29)	23.33(14.94,35.09)	79.83(15.36,179.40)	-32.94(-56.98,4.19)	-1.24(-1.53,-0.96)
Angola	65.71(36.09,103.89)	10.55(5.79,16.68)	216.92(128.76,327.19)	11.22(6.66,16.92)	230.12(114.78,403.57)	6.35(-30.81,62.22)	0.06(-0.01,0.13)
Antigua and Barbuda	1.61(1.46,1.78)	18.65(16.86,20.66)	1.77(1.60,2.00)	9.39(8.49,10.61)	9.92(-2.76,22.66)	-49.66(-55.47,-43.82)	-3.06(-3.39,-2.73)
Argentina	1482.96(1359.80,1626.53)	26.65(24.44,29.23)	1398.46(1273.65,1524.29)	14.98(13.65,16.33)	-5.70(-17.79,6.69)	-43.78(-50.99,-36.39)	-2.04(-2.32,-1.75)
Armenia	96.25(78.11,118.13)	19.77(16.05,24.27)	367.49(305.24,432.52)	46.72(38.80,54.98)	281.80(186.85,414.19)	136.26(77.51,218.18)	3.27(2.99,3.55)
Australia	1432.38(1326.41,1529.52)	43.65(40.42,46.61)	1143.49(990.52,1247.34)	15.44(13.37,16.84)	-20.17(-28.64,-13.02)	-64.64(-68.39,-61.47)	-4.01(-4.23,-3.79)
Austria	373.58(349.90,395.46)	19.20(17.98,20.32)	350.26(307.20,380.57)	11.85(10.40,12.88)	-6.24(-14.27,1.77)	-38.26(-43.55,-32.99)	-1.76(-1.94,-1.59)
Azerbaijan	26.87(19.97,35.75)	3.08(2.29,4.10)	107.54(70.39,167.80)	5.61(3.67,8.76)	300.30(138.19,530.88)	82.25(8.44,187.22)	2.56(2.07,3.05)
Bahamas	4.62(4.17,5.22)	18.83(16.99,21.25)	9.16(7.60,10.94)	12.73(10.56,15.20)	98.05(57.95,143.54)	-32.42(-46.10,-16.89)	-1.41(-1.78,-1.03)
Bahrain	0.66(0.54,0.86)	2.37(1.92,3.05)	3.50(2.66,4.75)	2.16(1.64,2.93)	427.68(236.99,660.67)	-8.82(-41.77,31.44)	-0.70(-1.13,-0.27)
Bangladesh	286.59(169.36,505.58)	3.78(2.24,6.67)	1306.39(842.36,2159.40)	5.59(3.61,9.24)	355.84(206.71,624.79)	47.77(-0.57,134.96)	1.25(1.08,1.43)
Barbados	8.68(7.97,9.45)	18.52(17.00,20.17)	11.14(9.03,13.64)	12.22(9.91,14.97)	28.28(3.35,58.72)	-34.00(-46.82,-18.33)	-2.31(-2.72,-1.90)
Belarus	277.78(239.88,338.05)	12.00(10.36,14.60)	515.10(422.92,615.33)	17.91(14.70,21.39)	85.44(44.80,137.37)	49.23(16.53,91.03)	1.00(0.59,1.42)
Belgium	785.31(723.95,844.45)	29.95(27.61,32.21)	586.53(497.33,638.40)	15.51(13.15,16.89)	-25.31(-33.42,-18.19)	-48.20(-53.83,-43.26)	-2.32(-2.53,-2.10)
Belize	0.84(0.73,0.98)	5.46(4.76,6.34)	2.06(1.79,2.34)	4.12(3.57,4.68)	145.76(98.21,196.44)	-24.47(-39.09,-8.90)	-1.66(-2.44,-0.87)
Benin	25.04(11.82,44.61)	7.83(3.69,13.94)	57.77(23.11,105.41)	6.99(2.79,12.75)	130.75(65.85,214.98)	-10.74(-35.85,21.84)	-0.48(-0.58,-0.39)
Bermuda	6.42(5.96,6.91)	60.70(56.38,65.31)	6.13(5.22,7.35)	26.30(22.40,31.54)	-4.60(-19.17,14.41)	-56.67(-63.29,-48.03)	-2.91(-3.06,-2.76)

location	1990		2021		1990-2021		
	Death cases	Death rate	Death cases	Death rate	Cases change	Rate change	EAPC
Bhutan	1.21(0.80,1.80)	3.04(2.02,4.53)	7.47(4.74,10.83)	7.50(4.76,10.87)	520.00(244.41,865.49)	147.06(37.24,284.74)	3.29(3.18,3.40)
Bolivia (Plurinational State of)	28.26(18.24,41.05)	5.39(3.48,7.83)	94.28(70.63,129.27)	6.10(4.57,8.36)	233.63(127.08,415.36)	13.19(-22.96,74.85)	0.52(0.47,0.57)
Bosnia and Herzegovina	66.10(48.10,90.73)	9.01(6.56,12.37)	177.58(127.20,235.51)	16.21(11.61,21.50)	168.64(67.43,330.04)	79.92(12.14,188.01)	2.30(2.03,2.56)
Botswana	8.94(5.73,14.01)	9.61(6.17,15.07)	20.54(12.77,27.79)	8.47(5.27,11.46)	129.83(39.19,299.01)	-11.86(-46.62,53.02)	-0.64(-0.92,-0.36)
Brazil	2209.81(2100.72,2297.39)	14.95(14.21,15.55)	8624.18(7841.36,9139.66)	19.91(18.11,21.10)	290.27(265.30,311.34)	33.18(24.66,40.37)	0.64(0.33,0.96)
Brunei Darussalam	4.01(3.16,4.95)	25.64(20.15,31.61)	10.27(8.53,12.29)	17.15(14.24,20.52)	155.80(91.23,249.47)	-33.11(-49.99,-8.62)	-1.13(-1.26,-1.00)
Bulgaria	178.91(158.17,198.64)	7.90(6.99,8.78)	325.79(265.91,402.54)	13.71(11.19,16.94)	82.10(41.99,135.07)	73.40(35.20,123.83)	1.57(1.23,1.90)
Burkina Faso	53.34(28.41,111.71)	7.37(3.93,15.45)	136.96(66.64,266.75)	9.11(4.43,17.74)	156.77(79.70,259.47)	23.52(-13.55,72.93)	0.79(0.72,0.87)
Burundi	41.42(23.00,66.36)	11.01(6.11,17.63)	44.66(19.18,83.40)	5.58(2.40,10.42)	7.83(-47.51,74.62)	-49.31(-75.32,-17.91)	-2.78(-3.12,-2.45)
Cabo Verde	4.11(1.62,8.59)	10.41(4.12,21.76)	8.92(4.38,15.83)	11.64(5.71,20.65)	117.09(49.86,239.16)	11.79(-22.83,74.65)	0.16(-0.11,0.43)
Cambodia	16.74(9.70,25.76)	2.26(1.31,3.48)	63.91(40.62,102.97)	2.94(1.87,4.74)	281.68(159.06,503.22)	30.09(-11.70,105.60)	0.92(0.85,0.98)
Cameroon	74.64(47.76,130.98)	10.08(6.45,17.69)	196.27(118.20,339.70)	9.64(5.81,16.69)	162.96(83.81,286.04)	-4.34(-33.13,40.43)	-0.43(-0.59,-0.27)
Canada	2059.75(1891.69,2247.84)	37.73(34.65,41.18)	1685.06(1454.97,1836.25)	13.73(11.86,14.96)	-18.19(-27.61,-9.54)	-63.61(-67.79,-59.76)	-4.06(-4.37,-3.74)
Central African Republic	19.57(10.89,34.26)	10.37(5.77,18.14)	28.09(14.50,50.36)	7.85(4.05,14.08)	43.53(-0.41,93.71)	-24.24(-47.43,2.25)	-1.13(-1.23,-1.03)
Chad	31.36(14.96,65.10)	6.77(3.23,14.05)	59.68(25.68,118.10)	6.43(2.77,12.72)	90.29(13.54,188.27)	-5.03(-43.33,43.87)	-0.42(-0.56,-0.29)
Chile	223.03(209.73,235.07)	13.26(12.47,13.98)	511.05(464.37,549.49)	11.41(10.37,12.27)	129.14(109.08,148.83)	-13.94(-21.47,-6.54)	-0.64(-0.99,-0.28)
China	1647.66(1307.92,2088.43)	1.15(0.91,1.46)	6743.41(5284.35,8574.60)	1.78(1.39,2.26)	309.27(175.62,488.90)	55.00(4.38,123.03)	1.60(1.49,1.72)
Colombia	468.99(441.59,496.99)	16.29(15.34,17.26)	1465.86(1221.25,1729.17)	15.33(12.77,18.08)	212.56(161.10,273.60)	-5.91(-21.40,12.47)	-1.35(-1.77,-0.93)
Comoros	3.17(1.21,5.82)	9.76(3.73,17.93)	6.96(2.63,12.81)	8.61(3.25,15.85)	119.67(39.79,225.98)	-11.80(-43.87,30.88)	-0.66(-0.85,-0.47)
Congo	29.50(18.58,43.71)	16.57(10.44,24.56)	58.05(34.50,88.25)	13.36(7.94,20.31)	96.79(41.87,170.56)	-19.38(-41.88,10.84)	-0.99(-1.14,-0.84)
Cook Islands	0.31(0.25,0.37)	14.35(11.75,17.41)	0.67(0.39,1.12)	14.17(8.19,23.86)	118.00(27.46,296.70)	-1.29(-42.29,79.62)	-0.08(-0.15,-0.01)
Costa Rica	34.41(31.21,38.24)	12.10(10.97,13.44)	117.35(101.81,132.25)	12.26(10.63,13.81)	241.00(188.93,295.04)	1.29(-14.18,17.34)	-0.48(-0.76,-0.20)
Croatia	61.48(29.33,110.56)	9.59(4.58,17.25)	174.16(78.43,313.20)	9.71(4.37,17.47)	123.76(77.70,177.57)	67.03(32.64,107.19)	-0.39(-0.62,-0.17)

location	1990		2021		1990-2021		
	Death cases	Death rate	Death cases	Death rate	Cases change	Rate change	EAPC
Cuba	135.49(117.20,157.04)	12.17(10.53,14.11)	303.18(258.38,349.08)	20.33(17.33,23.41)	37.55(18.68,61.70)	-31.78(-41.14,-19.81)	1.64(1.30,1.98)
Cyprus	537.99(492.96,581.51)	31.75(29.09,34.31)	740.03(628.29,834.80)	21.66(18.39,24.43)	63.75(11.49,141.16)	-34.55(-55.44,-3.61)	-1.52(-1.70,-1.34)
Czechia	47.18(36.38,60.80)	33.77(26.04,43.52)	77.26(58.05,97.75)	22.10(16.61,27.97)	66.30(42.51,90.74)	12.72(-3.41,29.28)	-2.01(-2.29,-1.73)
C 么 te d'Ivoire	359.94(334.23,388.33)	15.20(14.12,16.40)	598.59(520.47,679.33)	17.13(14.90,19.45)	183.25(99.92,295.83)	1.24(-28.54,41.48)	0.09(-0.28,0.46)
Democratic People's Republic of Korea	50.04(36.25,65.61)	1.88(1.36,2.47)	113.42(85.17,146.86)	2.01(1.51,2.61)	126.66(64.30,215.11)	7.04(-22.41,48.81)	0.68(0.48,0.89)
Democratic Republic of the Congo	262.27(130.69,467.39)	9.93(4.95,17.70)	514.41(256.15,917.19)	8.52(4.24,15.19)	96.14(39.87,177.49)	-14.25(-38.84,21.33)	-0.75(-0.95,-0.55)
Denmark	546.08(499.17,587.55)	41.86(38.26,45.04)	602.38(532.75,657.82)	31.29(27.67,34.17)	10.31(-1.33,22.71)	-25.25(-33.14,-16.84)	-1.59(-1.89,-1.30)
Djibouti	1.75(0.90,2.76)	8.15(4.19,12.88)	7.40(3.24,12.08)	7.13(3.12,11.65)	323.38(170.66,547.83)	-12.41(-44.01,34.02)	-0.72(-0.90,-0.55)
Dominica	2.16(1.65,2.75)	22.03(16.77,28.02)	2.93(2.36,3.63)	19.41(15.64,24.06)	35.32(-4.04,96.98)	-11.92(-37.53,28.22)	-0.84(-1.16,-0.53)
Dominican Republic	40.18(32.29,50.20)	6.57(5.28,8.21)	140.96(102.75,185.40)	8.44(6.15,11.10)	250.83(143.70,396.65)	28.36(-10.83,81.72)	0.53(0.22,0.83)
Ecuador	55.32(50.88,59.81)	6.43(5.92,6.96)	178.74(143.45,223.24)	6.46(5.18,8.07)	223.10(155.22,314.24)	0.38(-20.71,28.70)	0.55(0.14,0.95)
Egypt	61.29(42.59,90.23)	1.37(0.95,2.02)	183.62(144.08,230.76)	1.66(1.30,2.09)	199.59(87.87,358.44)	21.06(-24.09,85.24)	0.63(0.55,0.72)
El Salvador	12.51(10.38,15.00)	2.56(2.12,3.07)	29.12(22.73,37.05)	2.85(2.22,3.62)	132.75(66.91,212.34)	11.29(-20.19,49.35)	0.14(-0.01,0.30)
Equatorial Guinea	3.61(2.06,5.96)	11.32(6.46,18.68)	11.21(5.56,19.01)	14.17(7.03,24.04)	210.25(62.41,455.16)	25.21(-34.46,124.04)	0.83(0.77,0.90)
Eritrea	11.25(5.86,20.45)	6.36(3.32,11.57)	30.38(13.59,57.83)	6.90(3.08,13.12)	170.14(54.13,370.87)	8.35(-38.18,88.86)	-0.10(-0.24,0.05)
Estonia	50.04(45.76,54.98)	13.96(12.76,15.34)	92.17(80.37,104.23)	21.07(18.37,23.83)	84.19(57.56,116.96)	50.93(29.11,77.78)	1.00(0.68,1.33)
Eswatini	4.40(2.95,6.39)	9.74(6.53,14.15)	7.67(5.13,10.76)	8.47(5.66,11.88)	74.40(27.47,145.71)	-13.02(-36.43,22.54)	-0.47(-0.62,-0.32)
Ethiopia	152.76(88.06,278.25)	4.75(2.74,8.66)	342.42(175.70,581.98)	5.00(2.57,8.50)	124.16(44.43,262.76)	5.21(-32.21,70.26)	-0.05(-0.29,0.19)
Fiji	7.24(5.85,8.88)	12.85(10.38,15.76)	17.58(13.17,22.63)	12.85(9.63,16.55)	142.70(75.44,247.50)	0.04(-27.68,43.24)	-0.28(-0.40,-0.16)
Finland	536.12(496.57,572.05)	45.15(41.82,48.18)	501.40(433.34,548.05)	24.91(21.53,27.23)	-6.48(-16.22,3.32)	-44.83(-50.57,-39.05)	-2.04(-2.20,-1.88)
France	3043.48(2817.29,3219.33)	21.87(20.24,23.13)	3030.11(2601.80,3281.70)	13.70(11.77,14.84)	-0.44(-9.93,7.45)	-37.33(-43.31,-32.37)	-2.12(-2.42,-1.82)
Gabon	19.73(12.10,28.36)	20.15(12.35,28.97)	28.00(17.42,40.69)	15.67(9.75,22.76)	41.92(-3.50,97.58)	-22.27(-47.15,8.21)	-1.06(-1.21,-0.91)
Gambia	5.42(2.32,10.48)	9.80(4.18,18.93)	17.26(7.97,30.23)	11.16(5.16,19.55)	218.23(127.67,350.79)	13.96(-18.47,61.43)	0.30(0.15,0.44)

location	1990		2021		1990-2021		
	Death cases	Death rate	Death cases	Death rate	Cases change	Rate change	EAPC
Georgia	30.46(25.74,35.94)	2.74(2.32,3.24)	155.17(131.33,180.39)	14.78(12.51,17.18)	409.39(288.66,546.65)	438.92(311.20,584.15)	7.90(6.61,9.19)
Germany	4208.58(3824.46,4518.61)	20.06(18.23,21.53)	4483.27(3892.54,4877.78)	14.23(12.35,15.48)	6.53(-4.58,18.68)	-29.07(-36.46,-20.98)	-1.10(-1.21,-0.99)
Ghana	130.31(63.42,218.21)	12.76(6.21,21.37)	327.05(150.03,544.27)	11.99(5.50,19.96)	150.97(68.61,257.41)	-6.02(-36.86,33.84)	-0.48(-0.66,-0.30)
Greece	535.90(497.90,571.57)	20.04(18.62,21.38)	1015.42(905.85,1098.14)	27.63(24.65,29.88)	89.48(71.50,107.37)	37.83(24.76,50.85)	0.60(0.35,0.85)
Greenland	0.88(0.77,0.99)	15.89(13.89,18.05)	0.98(0.78,1.24)	7.18(5.73,9.15)	11.43(-13.57,45.82)	-54.77(-64.92,-40.82)	-2.59(-2.78,-2.40)
Grenada	3.51(3.07,4.21)	29.08(25.42,34.88)	4.36(3.81,4.89)	21.69(18.98,24.34)	24.23(-0.50,49.81)	-25.42(-40.27,-10.06)	-2.09(-2.66,-1.51)
Guam	3.09(2.61,3.66)	23.99(20.26,28.37)	2.97(2.48,3.51)	7.87(6.59,9.32)	-4.07(-25.38,23.20)	-67.18(-74.47,-57.85)	-3.64(-3.81,-3.48)
Guatemala	13.63(12.40,15.12)	2.37(2.16,2.63)	36.92(31.47,43.25)	2.02(1.72,2.36)	170.89(122.89,229.14)	-14.91(-29.98,3.39)	-1.02(-1.29,-0.75)
Guinea	44.41(21.10,85.29)	8.02(3.81,15.41)	78.67(28.91,153.28)	8.61(3.16,16.78)	77.15(2.58,159.48)	7.31(-37.86,57.18)	0.05(-0.15,0.25)
Guinea-Bissau	6.79(3.63,12.20)	10.64(5.69,19.12)	9.91(5.23,17.78)	8.62(4.56,15.47)	45.92(4.83,110.07)	-18.91(-41.75,16.74)	-0.92(-1.04,-0.81)
Guyana	5.87(5.25,6.47)	9.57(8.57,10.55)	17.26(13.33,21.79)	15.33(11.84,19.35)	194.14(120.11,274.15)	60.10(19.81,103.65)	0.97(0.23,1.71)
Haiti	53.24(34.84,80.98)	9.87(6.46,15.02)	113.47(70.80,176.07)	9.57(5.97,14.86)	113.12(53.60,191.94)	-3.01(-30.09,32.87)	-0.10(-0.22,0.03)
Honduras	12.10(8.91,16.67)	3.63(2.67,5.00)	58.74(41.75,79.24)	5.59(3.97,7.53)	385.36(239.09,653.51)	53.83(7.47,138.82)	1.50(1.35,1.66)
Hungary	405.54(371.26,440.29)	15.74(14.41,17.09)	528.83(464.57,596.08)	16.63(14.61,18.74)	30.40(13.35,48.91)	5.62(-8.19,20.61)	-0.18(-0.32,-0.03)
Iceland	12.37(11.08,13.27)	25.93(23.21,27.82)	16.13(13.53,18.07)	16.56(13.89,18.56)	30.33(15.04,46.66)	-36.15(-43.64,-28.15)	-1.99(-2.38,-1.59)
India	2145.30(1276.94,3504.88)	2.80(1.67,4.57)	11234.41(8054.26,16394.59)	5.59(4.01,8.15)	423.67(285.38,642.50)	99.70(46.96,183.16)	2.44(2.29,2.58)
Indonesia	405.43(265.40,551.13)	2.51(1.64,3.41)	1566.25(1074.87,2100.08)	3.74(2.57,5.01)	286.32(149.21,453.47)	49.03(-3.87,113.51)	1.15(0.91,1.38)
Iran (Islamic Republic of)	46.29(36.90,58.44)	1.00(0.80,1.27)	303.80(267.37,344.05)	2.34(2.06,2.65)	556.24(382.84,760.07)	132.87(71.34,205.20)	3.71(3.23,4.20)
Iraq	15.60(11.22,20.88)	1.24(0.89,1.65)	60.08(43.44,78.45)	1.55(1.12,2.02)	285.22(144.63,526.29)	25.16(-20.52,103.48)	0.58(0.46,0.69)
Ireland	254.08(236.82,272.13)	37.33(34.79,39.98)	237.80(197.68,264.77)	18.12(15.06,20.18)	-6.41(-19.28,5.24)	-51.45(-58.13,-45.41)	-2.85(-3.19,-2.50)
Israel	105.31(96.42,113.41)	12.99(11.90,13.99)	165.06(144.24,180.16)	8.26(7.22,9.01)	56.73(39.49,76.48)	-36.43(-43.43,-28.42)	-2.05(-2.33,-1.78)
Italy	2642.50(2472.76,2746.40)	17.36(16.25,18.05)	3507.33(3039.69,3789.43)	15.44(13.38,16.68)	32.73(21.80,40.76)	-11.08(-18.40,-5.70)	-0.92(-1.27,-0.58)
Jamaica	30.97(27.93,34.93)	10.51(9.48,11.86)	52.47(41.13,65.95)	9.92(7.78,12.47)	69.45(27.45,118.19)	-5.64(-29.02,21.51)	-0.40(-0.74,-0.06)

location	1990		2021		1990-2021		
	Death cases	Death rate	Death cases	Death rate	Cases change	Rate change	EAPC
Japan	4490.11(4141.27,4673.24)	15.16(13.98,15.78)	23012.10(18379.46,25666.31)	44.08(35.21,49.17)	412.51(340.19,453.08)	190.74(149.71,213.76)	3.65(3.55,3.75)
Jordan	9.66(7.30,12.78)	4.49(3.39,5.94)	47.12(35.87,61.22)	3.75(2.85,4.87)	387.76(212.75,652.04)	-16.58(-46.51,28.61)	-0.55(-0.84,-0.26)
Kazakhstan	135.63(108.66,173.29)	6.49(5.20,8.29)	311.79(249.89,380.29)	9.82(7.87,11.98)	129.88(62.94,223.81)	51.49(7.38,113.39)	0.58(0.22,0.94)
Kenya	84.61(53.94,123.46)	6.35(4.05,9.26)	269.52(158.48,376.16)	7.20(4.24,10.06)	218.54(133.06,307.94)	13.49(-16.97,45.34)	0.23(0.16,0.30)
Kiribati	0.09(0.08,0.11)	1.57(1.28,1.91)	0.20(0.14,0.26)	1.56(1.15,2.08)	109.75(41.22,191.91)	-0.64(-33.10,38.28)	-0.22(-0.35,-0.09)
Kuwait	3.49(3.12,3.88)	3.82(3.41,4.24)	15.62(12.84,19.29)	3.35(2.75,4.14)	347.23(256.83,464.84)	-12.29(-30.02,10.78)	0.11(-0.91,1.15)
Kyrgyzstan	6.14(5.15,7.41)	1.19(1.00,1.44)	27.00(21.41,33.24)	3.19(2.53,3.93)	339.47(223.95,496.73)	167.56(97.23,263.31)	3.86(3.24,4.49)
Lao People's Democratic Republic	10.77(6.92,16.77)	3.14(2.02,4.89)	28.25(20.83,39.14)	3.65(2.69,5.06)	162.31(76.39,306.69)	16.35(-21.76,80.39)	0.46(0.38,0.54)
Latvia	69.35(63.67,75.64)	11.09(10.18,12.10)	114.93(98.44,129.98)	17.47(14.96,19.75)	65.71(42.25,93.20)	57.46(35.16,83.57)	1.18(0.89,1.47)
Lebanon	44.75(21.86,82.00)	11.80(5.77,21.63)	120.56(96.58,151.31)	12.30(9.85,15.44)	169.42(36.04,503.68)	4.23(-47.37,133.54)	0.44(0.16,0.71)
Lesotho	10.18(5.84,16.13)	7.28(4.18,11.54)	14.04(7.61,22.12)	7.78(4.21,12.25)	37.89(-6.66,100.80)	6.80(-27.71,55.52)	0.19(0.02,0.35)
Liberia	19.41(10.11,35.40)	10.30(5.36,18.78)	26.62(11.57,50.74)	8.06(3.50,15.37)	37.12(-8.74,89.89)	-21.73(-47.91,8.39)	-0.92(-0.99,-0.84)
Libya	1.53(1.09,2.22)	0.49(0.35,0.72)	11.35(7.16,17.39)	1.36(0.86,2.08)	640.20(378.57,1071.41)	175.28(77.98,335.66)	4.19(3.65,4.73)
Lithuania	81.59(74.70,89.64)	10.30(9.43,11.31)	170.66(148.76,193.18)	17.63(15.37,19.96)	109.16(80.60,141.86)	71.27(47.88,98.04)	1.74(1.53,1.95)
Luxembourg	22.64(21.11,24.34)	24.25(22.61,26.06)	24.67(21.70,27.36)	13.88(12.21,15.39)	8.96(-3.46,22.18)	-42.74(-49.26,-35.79)	-2.22(-2.49,-1.95)
Madagascar	108.81(54.32,190.77)	12.88(6.43,22.58)	165.19(80.03,282.63)	8.95(4.33,15.30)	51.81(8.63,114.13)	-30.54(-50.30,-2.03)	-1.43(-1.60,-1.25)
Malawi	38.44(18.48,73.06)	6.11(2.94,11.62)	91.85(45.00,159.35)	7.86(3.85,13.63)	138.93(71.60,237.16)	28.54(-7.68,81.39)	0.46(0.26,0.65)
Malaysia	237.29(190.49,289.58)	16.08(12.91,19.62)	887.69(739.12,1072.42)	18.11(15.08,21.87)	274.10(177.67,408.97)	12.61(-16.41,53.22)	-0.09(-0.34,0.17)
Maldives	0.35(0.24,0.49)	2.27(1.56,3.19)	1.41(0.73,2.26)	2.59(1.35,4.15)	304.78(140.94,552.90)	14.07(-32.10,84.00)	0.22(-0.01,0.44)
Mali	33.25(16.62,67.20)	4.97(2.49,10.05)	71.10(29.75,140.83)	4.91(2.06,9.73)	113.84(22.86,256.66)	-1.21(-43.24,64.77)	0.09(-0.14,0.31)
Malta	10.31(9.54,11.15)	14.24(13.17,15.38)	13.52(11.84,15.18)	8.57(7.50,9.62)	31.13(14.42,49.34)	-39.80(-47.47,-31.44)	-2.46(-2.82,-2.11)
Marshall Islands	0.30(0.20,0.44)	11.94(7.95,17.08)	0.54(0.37,0.77)	9.28(6.30,13.08)	78.60(32.34,146.10)	-22.25(-42.39,7.13)	-1.32(-1.48,-1.16)
Mauritania	21.11(9.63,34.41)	12.72(5.80,20.73)	38.76(14.19,68.10)	10.80(3.95,18.98)	83.61(8.97,179.60)	-15.03(-49.57,29.38)	-0.99(-1.17,-0.82)

location	1990		2021		1990-2021		
	Death cases	Death rate	Death cases	Death rate	Cases change	Rate change	EAPC
Mauritius	7.77(7.26,8.27)	6.39(5.97,6.80)	13.52(12.33,14.40)	4.00(3.65,4.26)	73.95(58.10,88.67)	-37.46(-43.16,-32.17)	-1.59(-2.01,-1.16)
Mexico	242.40(233.76,249.98)	3.49(3.36,3.60)	643.82(568.73,726.14)	2.99(2.64,3.37)	165.60(136.84,197.95)	-14.31(-23.59,-3.87)	-0.83(-1.01,-0.64)
Micronesia (Federated States of)	1.29(0.97,1.74)	16.16(12.17,21.86)	1.50(1.12,1.96)	11.42(8.55,14.92)	16.82(-14.35,65.61)	-29.32(-48.18,0.19)	-1.53(-1.66,-1.40)
Monaco	4.34(3.23,5.41)	39.13(29.17,48.86)	5.70(4.30,7.63)	36.41(27.49,48.78)	31.36(-7.44,95.50)	-6.96(-34.44,38.48)	-0.26(-0.47,-0.05)
Mongolia	2.47(1.71,3.39)	1.43(0.99,1.96)	10.64(8.03,13.59)	2.70(2.03,3.44)	330.66(185.66,571.76)	89.02(25.38,194.84)	2.29(1.96,2.62)
Montenegro	34.21(26.56,44.94)	31.40(24.38,41.25)	74.90(56.67,98.81)	42.96(32.50,56.66)	118.93(54.07,224.20)	36.78(-3.74,102.55)	1.44(1.30,1.58)
Morocco	13.09(8.09,17.96)	0.56(0.34,0.77)	113.96(78.98,147.79)	1.90(1.31,2.46)	770.46(528.16,1111.21)	239.73(145.16,372.72)	4.67(4.42,4.93)
Mozambique	74.59(32.79,146.91)	7.63(3.36,15.03)	173.82(72.90,335.52)	9.72(4.07,18.75)	133.02(60.02,215.35)	27.27(-12.60,72.24)	0.85(0.78,0.93)
Myanmar	125.06(73.19,186.46)	3.19(1.87,4.76)	340.17(255.68,447.27)	4.03(3.03,5.30)	172.00(82.05,321.54)	26.12(-15.59,95.46)	0.70(0.56,0.85)
Namibia	10.63(6.91,16.19)	9.79(6.36,14.92)	22.48(16.45,30.67)	9.95(7.28,13.57)	111.56(52.02,208.30)	1.60(-26.99,48.06)	-0.20(-0.40,-0.01)
Nauru	0.14(0.10,0.18)	18.97(14.25,24.84)	0.19(0.12,0.27)	20.56(12.67,29.60)	38.56(-8.91,101.03)	8.35(-28.77,57.20)	0.01(-0.17,0.20)
Nepal	40.46(22.58,72.89)	2.63(1.47,4.73)	209.88(143.29,327.23)	5.30(3.62,8.27)	418.69(244.27,692.09)	101.73(33.90,208.07)	2.53(2.34,2.72)
Netherlands	1486.37(1366.78,1595.45)	44.89(41.28,48.19)	1425.24(1223.77,1563.79)	24.36(20.92,26.73)	-4.11(-13.23,5.06)	-45.74(-50.90,-40.55)	-2.70(-3.06,-2.33)
New Zealand	394.34(364.97,419.78)	59.93(55.47,63.80)	348.80(304.53,378.24)	24.45(21.35,26.52)	-11.55(-20.52,-3.55)	-59.20(-63.34,-55.51)	-3.53(-3.76,-3.30)
Nicaragua	3.93(3.30,4.65)	1.58(1.33,1.87)	13.51(10.60,16.85)	1.66(1.30,2.07)	243.77(155.45,347.88)	4.93(-22.03,36.71)	0.09(-0.24,0.43)
Niger	23.48(9.92,49.10)	5.34(2.26,11.17)	64.15(23.76,148.57)	4.61(1.71,10.67)	173.23(73.01,282.77)	-13.75(-45.38,20.83)	-0.69(-0.84,-0.55)
Nigeria	835.81(424.81,1539.94)	11.47(5.83,21.14)	1384.28(656.09,2494.85)	9.23(4.37,16.63)	65.62(13.68,127.82)	-19.57(-44.79,10.63)	-1.02(-1.19,-0.85)
Niue	0.08(0.06,0.10)	22.21(17.53,27.53)	0.06(0.05,0.07)	15.07(11.72,18.50)	-27.10(-45.32,-4.87)	-32.15(-49.11,-11.46)	-1.21(-1.27,-1.15)
North Macedonia	28.96(22.44,36.46)	8.80(6.82,11.08)	73.81(48.32,109.85)	12.45(8.15,18.53)	154.88(60.91,283.70)	41.42(-10.72,112.89)	0.98(0.82,1.15)
Northern Mariana Islands	0.43(0.34,0.53)	17.52(13.99,21.90)	1.13(0.90,1.42)	11.59(9.26,14.64)	163.42(90.13,276.29)	-33.84(-52.25,-5.49)	-2.76(-3.19,-2.33)
Norway	556.60(522.60,582.11)	51.40(48.26,53.75)	530.21(455.96,570.32)	32.69(28.12,35.17)	-4.74(-12.01,0.90)	-36.39(-41.25,-32.62)	-2.25(-2.64,-1.87)
Oman	0.76(0.48,1.21)	0.76(0.48,1.21)	7.00(4.19,11.29)	2.24(1.34,3.62)	819.70(276.69,1985.87)	195.94(21.21,571.19)	4.27(3.75,4.80)
Pakistan	366.52(248.83,560.05)	4.02(2.73,6.14)	1207.16(870.02,1648.37)	6.09(4.39,8.31)	229.35(131.91,360.08)	51.49(6.67,111.63)	1.07(0.75,1.38)

location	1990		2021		1990-2021		
	Death cases	Death rate	Death cases	Death rate	Cases change	Rate change	EAPC
Palau	0.21(0.16,0.27)	13.13(10.04,16.60)	0.39(0.30,0.52)	9.43(7.18,12.47)	87.96(34.03,169.15)	-28.17(-48.78,2.86)	-1.35(-1.49,-1.22)
Palestine	4.02(2.87,5.37)	2.77(1.98,3.70)	13.28(10.08,17.02)	3.09(2.34,3.95)	229.88(120.15,402.75)	11.28(-25.73,69.60)	0.49(0.36,0.62)
Panama	28.06(25.40,30.52)	11.58(10.48,12.60)	70.01(53.54,84.86)	9.42(7.20,11.41)	149.46(93.19,203.09)	-18.71(-37.05,-1.24)	-1.18(-1.42,-0.93)
Papua New Guinea	15.79(10.00,24.63)	5.34(3.38,8.32)	45.90(30.51,67.69)	5.59(3.71,8.24)	190.67(100.76,345.55)	4.72(-27.68,60.51)	-0.10(-0.23,0.03)
Paraguay	36.09(29.17,44.81)	9.95(8.04,12.35)	139.90(106.15,180.26)	14.13(10.72,18.21)	287.65(179.34,451.02)	42.03(2.35,101.89)	1.20(1.05,1.35)
Peru	72.48(56.63,93.73)	3.68(2.87,4.75)	187.02(137.05,248.66)	3.34(2.45,4.45)	158.02(75.08,278.97)	-9.05(-38.28,33.59)	-0.27(-0.40,-0.14)
Philippines	232.39(194.48,274.38)	4.87(4.07,5.75)	779.27(639.70,914.90)	5.59(4.59,6.56)	235.33(171.85,329.86)	14.80(-6.93,47.16)	0.33(0.23,0.42)
Poland	1743.51(1674.71,1796.37)	22.63(21.73,23.31)	2422.15(2169.39,2662.28)	19.99(17.91,21.97)	38.92(26.66,52.95)	-11.64(-19.44,-2.71)	-1.06(-1.29,-0.82)
Portugal	218.23(203.98,232.38)	8.95(8.37,9.53)	385.66(342.64,416.62)	9.91(8.81,10.71)	76.72(59.52,93.90)	10.74(-0.04,21.50)	0.02(-0.16,0.20)
Puerto Rico	75.71(70.18,80.61)	12.48(11.57,13.28)	74.19(60.68,86.92)	6.36(5.20,7.45)	-2.01(-18.51,15.61)	-49.07(-57.64,-39.91)	-3.06(-3.35,-2.76)
Qatar	0.76(0.60,0.97)	4.64(3.66,5.94)	4.33(2.41,7.61)	2.83(1.57,4.97)	471.23(174.37,1052.25)	-39.09(-70.74,22.87)	-2.06(-2.51,-1.60)
Republic of Korea	354.28(237.79,499.76)	7.12(4.78,10.04)	1669.97(1347.70,1975.45)	9.99(8.06,11.81)	371.37(191.21,679.10)	40.27(-13.34,131.85)	1.09(0.83,1.34)
Republic of Moldova	31.51(28.27,34.86)	4.08(3.66,4.52)	79.11(69.79,89.16)	7.49(6.61,8.44)	151.05(113.05,200.11)	83.57(55.78,119.44)	1.83(1.51,2.14)
Romania	294.66(270.01,321.32)	5.89(5.40,6.42)	623.99(542.78,717.85)	10.39(9.04,11.95)	111.76(80.32,147.98)	76.45(50.25,106.62)	1.59(1.40,1.77)
Russian Federation	3887.51(3750.36,3980.56)	12.32(11.88,12.61)	9308.95(8507.03,10081.25)	21.90(20.01,23.72)	139.46(120.49,157.82)	77.81(63.73,91.45)	1.55(1.07,2.02)
Rwanda	51.63(30.37,81.68)	11.03(6.49,17.45)	76.75(45.86,127.71)	7.26(4.34,12.08)	48.64(-9.06,144.25)	-34.22(-59.76,8.09)	-2.28(-2.70,-1.86)
Saint Kitts and Nevis	1.35(1.22,1.50)	21.18(19.11,23.48)	1.28(1.05,1.58)	9.96(8.18,12.24)	-5.29(-23.45,16.98)	-52.98(-61.99,-41.92)	-3.10(-3.63,-2.56)
Saint Lucia	7.31(6.64,8.17)	49.93(45.37,55.83)	10.75(9.05,12.53)	25.55(21.51,29.78)	47.17(21.45,76.36)	-48.84(-57.78,-38.69)	-2.90(-3.23,-2.57)
Saint Vincent and the Grenadines	1.65(1.52,1.81)	13.75(12.66,15.04)	2.59(2.29,2.92)	10.19(9.02,11.50)	56.86(35.81,84.40)	-25.88(-35.83,-12.86)	-1.55(-1.98,-1.12)
Samoa	1.99(1.40,2.65)	13.78(9.74,18.36)	2.90(2.21,3.71)	11.77(8.99,15.06)	45.96(13.94,93.51)	-14.56(-33.30,13.27)	-0.58(-0.64,-0.52)
San Marino	1.10(0.88,1.35)	18.79(14.98,22.96)	1.42(0.92,2.20)	12.02(7.80,18.62)	28.89(-17.92,100.66)	-36.00(-59.24,-0.36)	-0.23(-0.74,0.29)
Sao Tome and Principe	0.93(0.44,1.69)	8.28(3.94,15.11)	1.76(0.89,3.10)	9.63(4.88,16.94)	89.97(40.04,153.65)	16.35(-14.23,55.35)	0.65(0.52,0.77)
Saudi Arabia	2.82(1.97,3.99)	0.32(0.22,0.45)	19.97(14.77,27.32)	0.66(0.49,0.91)	609.28(355.40,1013.16)	109.88(34.76,229.40)	3.14(2.69,3.59)

location	1990		2021		1990-2021		
	Death cases	Death rate	Death cases	Death rate	Cases change	Rate change	EAPC
Senegal	51.49(24.23,95.71)	9.74(4.59,18.11)	121.23(50.56,223.81)	9.39(3.92,17.33)	135.43(67.74,215.11)	-3.65(-31.35,28.96)	-0.40(-0.51,-0.28)
Serbia	349.83(290.10,418.50)	16.82(13.95,20.12)	652.48(518.59,820.94)	23.26(18.49,29.26)	86.51(40.83,147.56)	38.28(4.41,83.54)	1.22(1.04,1.40)
Seychelles	0.55(0.44,0.66)	5.69(4.60,6.88)	0.95(0.65,1.34)	4.59(3.14,6.48)	74.47(25.21,141.53)	-19.32(-42.10,11.69)	-0.62(-0.97,-0.26)
Sierra Leone	35.09(16.78,65.53)	10.59(5.06,19.77)	51.11(22.37,99.60)	8.46(3.70,16.49)	45.67(1.55,111.72)	-20.07(-44.28,16.17)	-1.02(-1.13,-0.91)
Singapore	45.62(42.92,48.06)	12.58(11.84,13.25)	164.89(145.59,178.52)	10.86(9.59,11.75)	261.43(222.98,296.89)	-13.72(-22.90,-5.26)	-0.60(-0.80,-0.40)
Slovakia	116.44(99.92,137.37)	11.22(9.63,13.24)	197.13(155.30,249.16)	12.02(9.47,15.19)	69.30(21.95,133.11)	7.08(-22.87,47.44)	0.05(-0.11,0.20)
Slovenia	57.61(52.92,61.99)	13.37(12.29,14.39)	100.93(84.85,121.43)	13.78(11.59,16.59)	75.21(47.02,113.09)	3.07(-13.52,25.35)	-0.10(-0.45,0.25)
Solomon Islands	1.31(0.85,2.12)	5.78(3.77,9.38)	3.78(2.57,5.51)	6.96(4.74,10.16)	189.03(102.26,297.64)	20.46(-15.70,65.73)	0.63(0.54,0.72)
Somalia	16.92(8.80,34.72)	4.70(2.44,9.64)	30.91(14.45,73.78)	3.28(1.53,7.82)	82.68(19.88,171.72)	-30.25(-54.23,3.75)	-1.39(-1.67,-1.11)
South Africa	461.88(347.83,579.91)	13.68(10.30,17.18)	762.90(676.06,849.75)	9.67(8.57,10.77)	65.17(33.77,120.72)	-29.32(-42.76,-5.55)	-1.88(-2.31,-1.46)
South Sudan	38.20(15.20,70.69)	9.21(3.67,17.05)	33.91(14.78,66.18)	5.44(2.37,10.63)	-11.25(-38.46,29.53)	-40.90(-59.02,-13.74)	-2.11(-2.29,-1.92)
Spain	1303.87(1218.51,1380.31)	13.64(12.75,14.44)	2157.83(1885.34,2334.57)	13.94(12.18,15.09)	65.49(50.68,78.77)	2.24(-6.91,10.45)	-0.58(-0.96,-0.19)
Sri Lanka	25.07(20.78,30.63)	1.41(1.17,1.72)	80.91(55.34,113.94)	1.68(1.15,2.37)	222.72(101.43,411.94)	19.26(-25.56,89.18)	0.76(0.58,0.94)
Sudan	6.35(3.49,14.41)	0.43(0.23,0.97)	43.51(28.03,63.20)	1.40(0.90,2.03)	585.73(233.91,1151.65)	227.48(59.46,497.74)	4.49(4.19,4.79)
Suriname	4.93(4.12,5.89)	11.33(9.47,13.55)	11.01(7.62,15.34)	9.85(6.83,13.73)	123.18(47.06,216.73)	-13.05(-42.71,23.39)	-0.43(-0.64,-0.22)
Sweden	1183.96(1100.32,1251.95)	49.87(46.35,52.73)	963.24(825.60,1074.02)	28.61(24.52,31.90)	-18.64(-27.01,-10.19)	-42.63(-48.53,-36.66)	-2.36(-2.67,-2.05)
Switzerland	512.24(459.25,555.95)	30.33(27.19,32.91)	474.90(399.47,527.06)	16.20(13.63,17.98)	-7.29(-17.80,4.48)	-46.57(-52.63,-39.78)	-2.07(-2.26,-1.89)
Syrian Arab Republic	12.63(8.72,17.12)	1.43(0.99,1.94)	39.24(28.62,52.68)	1.66(1.21,2.23)	210.73(97.00,395.26)	15.95(-26.49,84.81)	0.36(0.26,0.45)
Taiwan (Province of China)	152.01(141.55,162.38)	5.48(5.10,5.85)	842.65(743.85,918.26)	11.19(9.88,12.19)	454.33(395.52,513.09)	104.18(82.52,125.83)	1.28(0.44,2.13)
Tajikistan	5.85(3.94,7.77)	1.25(0.84,1.66)	9.64(6.96,13.31)	0.94(0.68,1.30)	64.83(12.00,149.57)	-24.85(-48.93,13.79)	-0.95(-1.49,-0.41)
Thailand	460.51(345.55,623.04)	7.76(5.82,10.50)	1960.65(1488.79,2558.16)	10.09(7.66,13.16)	325.75(187.29,521.58)	29.98(-12.29,89.77)	0.48(0.34,0.62)
Timor-Leste	0.85(0.61,1.29)	1.96(1.40,2.96)	4.74(3.11,7.42)	3.34(2.19,5.22)	456.72(267.56,719.13)	70.36(12.47,150.65)	1.89(1.71,2.06)
Togo	19.79(9.59,36.41)	10.15(4.91,18.67)	56.51(23.87,101.68)	9.05(3.82,16.28)	185.50(101.08,278.47)	-10.85(-37.21,18.19)	-0.71(-0.85,-0.58)

location	1990		2021		1990-2021		
	Death cases	Death rate	Death cases	Death rate	Cases change	Rate change	EAPC
Tokelau	0.04(0.03,0.05)	15.44(12.00,19.55)	0.05(0.03,0.07)	18.05(11.44,25.79)	24.86(-24.16,89.16)	16.91(-28.99,77.12)	0.31(0.09,0.53)
Tonga	1.16(0.90,1.49)	12.21(9.49,15.69)	2.04(1.45,2.70)	15.22(10.77,20.12)	76.52(14.68,154.51)	24.72(-18.97,79.82)	0.64(0.40,0.89)
Trinidad and Tobago	37.51(34.55,41.13)	27.32(25.16,29.96)	68.94(53.32,87.30)	19.79(15.31,25.06)	83.82(39.25,135.39)	-27.55(-45.11,-7.22)	-1.82(-2.20,-1.44)
Tunisia	5.43(3.74,7.44)	0.62(0.43,0.85)	46.30(30.02,67.54)	1.98(1.28,2.89)	751.90(431.35,1279.48)	217.38(97.96,413.94)	4.20(3.77,4.63)
Turkey	514.45(371.09,728.65)	8.61(6.21,12.20)	1727.26(1344.37,2173.25)	10.45(8.14,13.15)	280.68(152.22,456.22)	74.70(15.75,155.25)	0.63(0.47,0.79)
Turkmenistan	14.20(11.63,17.40)	4.37(3.58,5.36)	54.04(40.58,77.00)	7.64(5.73,10.88)	68.44(23.67,144.01)	7.39(-21.15,55.57)	1.70(1.43,1.97)
Tuvalu	0.13(0.08,0.18)	10.98(7.30,15.53)	0.21(0.17,0.27)	11.79(9.46,14.96)	235.75(115.23,402.10)	21.42(-22.17,81.57)	-0.07(-0.24,0.10)
Uganda	71.87(33.77,138.74)	6.87(3.23,13.27)	157.70(77.90,262.87)	6.65(3.28,11.08)	119.43(47.18,231.21)	-3.22(-35.09,46.07)	-0.35(-0.48,-0.23)
Ukraine	1242.61(1121.17,1385.91)	9.97(8.99,11.12)	1561.40(1179.08,2013.21)	11.50(8.68,14.83)	25.65(-10.10,67.48)	15.37(-17.45,53.78)	0.15(-0.17,0.47)
United Arab Emirates	2.91(1.78,4.36)	5.18(3.16,7.76)	23.47(18.55,30.67)	3.27(2.58,4.27)	706.60(420.34,1317.47)	-36.96(-59.33,10.79)	-0.16(-0.84,0.52)
United Kingdom	9338.55(8800.54,9610.76)	62.87(59.25,64.71)	5925.16(5226.43,6287.04)	28.17(24.85,29.90)	-36.55(-40.94,-34.25)	-55.19(-58.28,-53.56)	-3.18(-3.56,-2.79)
United Republic of Tanzania	173.38(103.26,287.67)	9.61(5.72,15.94)	454.19(220.76,788.46)	11.02(5.36,19.13)	161.96(54.49,354.13)	14.65(-32.38,98.76)	0.13(-0.00,0.26)
United States of America	16478.81(15192.02,17190.81)	31.41(28.96,32.77)	11017.55(9736.24,11744.76)	10.99(9.71,11.72)	-33.14(-36.27,-30.67)	-65.01(-66.65,-63.72)	-4.26(-4.59,-3.92)
United States Virgin Islands	2.03(1.66,2.43)	14.51(11.85,17.37)	3.47(2.63,4.39)	10.77(8.17,13.63)	71.08(22.77,138.18)	-25.77(-46.73,3.35)	-1.35(-1.67,-1.02)
Uruguay	208.47(194.01,225.50)	30.89(28.75,33.42)	239.70(217.60,258.80)	26.51(24.06,28.62)	14.98(2.64,27.07)	-14.20(-23.41,-5.18)	-0.90(-1.21,-0.59)
Uzbekistan	18.58(13.45,27.06)	0.95(0.69,1.38)	190.77(152.48,235.80)	4.10(3.28,5.07)	926.56(560.54,1421.79)	331.87(177.89,540.22)	5.45(4.79,6.11)
Vanuatu	0.80(0.55,1.16)	7.98(5.49,11.66)	2.22(1.63,2.97)	7.75(5.69,10.36)	179.23(108.90,276.23)	-2.94(-27.39,30.77)	-0.43(-0.58,-0.29)
Venezuela (Bolivarian Republic of)	171.91(159.28,185.70)	10.98(10.17,11.86)	473.24(373.16,589.76)	9.04(7.13,11.27)	175.29(111.70,249.19)	-17.61(-36.65,4.50)	-1.33(-1.71,-0.94)
Viet Nam	213.20(151.93,290.57)	3.06(2.18,4.17)	803.77(588.02,1063.49)	4.60(3.37,6.09)	277.00(158.00,482.55)	50.47(2.97,132.51)	1.47(1.13,1.80)
Yemen	3.27(1.73,5.78)	0.41(0.22,0.72)	30.87(18.26,48.31)	1.37(0.81,2.15)	845.18(510.92,1454.63)	237.07(117.87,454.42)	4.56(4.17,4.96)
Zambia	42.30(27.06,63.93)	9.19(5.88,13.89)	150.11(67.09,264.65)	13.90(6.21,24.50)	254.87(94.19,520.72)	51.23(-17.24,164.53)	1.54(1.18,1.91)
Zimbabwe	90.47(72.29,109.81)	13.65(10.91,16.57)	167.77(127.14,222.80)	15.15(11.48,20.12)	85.44(29.96,165.41)	11.00(-22.21,58.88)	0.04(-0.28,0.36)
