# Supplementary material for: The global burden of aortic aneurysm in adults over 55: evolving trends, risk factors, and projections
Source: Front Cardiovasc Med. 2025 Nov 3;12:1629635. doi: 10.3389/fcvm.2025.1629635 (PMC12620971; doi:10.3389/fcvm.2025.1629635)
Supplement: Supplementary file 2 [file Table2.pdf]

Table S2. DALYs of Aortic Aneurysm between 1990 and 2021 at national level.

location	1990		2021		1990-2021		
	DALY cases	DALY rate	DALY cases	DALY rate	Cases change	Rate change	EAPC
Afghanistan	97.93(50.98,170.29)	8.06(4.20,14.01)	338.60(217.11,463.95)	27.56(17.67,37.76)	245.76(131.34,461.19)	241.95(128.79,454.99)	4.99(4.65,5.34)
Albania	283.12(231.56,341.28)	81.58(66.73,98.34)	798.59(506.44,1170.12)	101.56(64.41,148.81)	182.07(75.77,352.58)	24.49(-22.42,99.74)	0.83(0.67,0.98)
Algeria	245.20(172.47,334.74)	11.70(8.23,15.98)	1901.86(1392.50,2534.92)	31.31(22.93,41.74)	675.62(420.68,1044.25)	167.55(79.61,294.71)	3.86(3.49,4.23)
American Samoa	10.45(8.76,12.40)	285.86(239.63,339.13)	17.80(14.74,21.74)	210.73(174.50,257.35)	70.27(32.37,117.18)	-26.28(-42.69,-5.97)	-1.35(-1.54,-1.15)
Andorra	61.76(39.32,91.53)	625.89(398.49,927.63)	96.45(60.52,149.22)	364.50(228.71,563.91)	56.18(-2.66,145.28)	-41.76(-63.70,-8.54)	-1.71(-1.97,-1.46)
Angola	1487.21(806.86,2349.85)	238.75(129.53,377.24)	4756.97(2840.39,7210.53)	246.01(146.89,372.90)	219.86(104.07,398.60)	3.04(-34.26,60.62)	-0.07(-0.16,0.01)
Antigua and Barbuda	27.43(24.85,30.39)	317.40(287.59,351.71)	32.84(29.54,37.63)	174.04(156.56,199.43)	19.73(5.71,34.03)	-45.17(-51.59,-38.62)	-2.78(-3.10,-2.45)
Argentina	29230.99(26692.75,32351.42)	525.30(479.69,581.38)	25922.38(23652.26,28302.66)	277.74(253.42,303.24)	-11.32(-23.49,1.15)	-47.13(-54.39,-39.70)	-2.27(-2.53,-2.01)
Armenia	2089.56(1697.01,2544.19)	429.27(348.63,522.67)	6905.43(5735.48,8145.63)	877.86(729.13,1035.52)	230.47(148.98,346.31)	104.50(54.07,176.18)	2.55(2.32,2.77)
Australia	25199.04(23453.32,26987.03)	767.90(714.71,822.39)	16997.35(15006.24,18361.06)	229.45(202.57,247.86)	-32.55(-39.23,-26.58)	-70.12(-73.08,-67.48)	-4.57(-4.78,-4.35)
Austria	6573.73(6187.58,6971.34)	337.82(317.98,358.26)	5572.78(4956.92,6002.83)	188.58(167.74,203.13)	-15.23(-22.25,-7.51)	-44.18(-48.80,-39.10)	-2.05(-2.22,-1.87)
Azerbaijan	579.80(435.11,774.96)	66.47(49.89,88.85)	2462.10(1455.31,4122.94)	128.52(75.96,215.21)	324.65(130.94,627.70)	93.33(5.14,231.30)	2.48(2.10,2.86)
Bahamas	90.72(81.87,102.92)	369.44(333.42,419.14)	175.87(145.18,211.99)	244.41(201.76,294.60)	93.87(52.76,141.23)	-33.84(-47.87,-17.68)	-1.45(-1.81,-1.08)
Bahrain	14.95(12.17,19.28)	53.27(43.38,68.73)	78.66(59.06,107.10)	48.45(36.37,65.96)	426.29(236.43,675.75)	-9.06(-41.87,34.05)	-0.63(-1.01,-0.25)
Bangladesh	5473.03(3123.41,10118.93)	72.25(41.23,133.59)	23519.85(15283.13,40305.92)	100.66(65.41,172.50)	329.74(184.08,601.68)	39.31(-7.91,127.47)	1.13(1.01,1.25)
Barbados	143.79(131.49,157.30)	306.69(280.45,335.50)	187.31(149.00,232.70)	205.57(163.52,255.39)	30.27(3.56,63.84)	-32.97(-46.71,-15.70)	-2.13(-2.49,-1.76)
Belarus	5894.03(5115.00,7201.38)	254.61(220.96,311.08)	10880.70(8895.31,13217.95)	378.25(309.23,459.50)	84.61(41.65,135.83)	48.56(14.00,89.79)	0.84(0.42,1.27)
Belgium	13515.78(12500.32,14530.71)	515.49(476.76,554.20)	8891.25(7802.12,9627.63)	235.17(206.36,254.65)	-34.22(-40.62,-27.40)	-54.38(-58.82,-49.65)	-2.83(-3.05,-2.60)
Belize	15.10(13.22,17.60)	98.09(85.87,114.36)	38.45(33.24,43.71)	76.76(66.37,87.28)	154.63(102.81,210.46)	-21.75(-37.67,-4.59)	-1.57(-2.34,-0.80)
Benin	476.03(224.03,857.22)	148.79(70.02,267.94)	1127.14(444.61,2073.36)	136.28(53.76,250.69)	136.78(65.80,225.77)	-8.41(-35.87,26.01)	-0.44(-0.56,-0.32)
Bermuda	115.94(108.11,125.10)	1096.02(1021.94,1182.58)	95.27(81.17,115.18)	409.03(348.53,494.52)	-17.83(-30.64,-1.60)	-62.68(-68.50,-55.31)	-3.37(-3.53,-3.22)

Bhutan	25.10(16.45,37.92)	63.24(41.44,95.56)	131.26(82.94,194.05)	131.80(83.28,194.85)	423.04(188.03,737.02)	108.42(14.78,233.54)	2.61(2.53,2.70)
Bolivia (Plurinational State of)	565.20(362.96,834.40)	107.79(69.22,159.14)	1812.08(1318.02,2494.06)	117.25(85.28,161.38)	220.61(110.50,415.70)	8.77(-28.58,74.96)	0.31(0.27,0.35)
Bosnia and Herzegovina	1418.42(1027.91,1957.23)	193.37(140.13,266.82)	3413.75(2457.37,4629.38)	311.68(224.36,422.67)	140.67(47.36,297.76)	61.19(-1.31,166.40)	1.77(1.54,2.00)
Botswana	185.81(118.19,297.02)	199.86(127.12,319.48)	396.61(243.80,535.62)	163.60(100.57,220.94)	113.44(29.21,271.57)	-18.14(-50.45,42.50)	-0.96(-1.23,-0.70)
Brazil	47531.56(45445.63,49251.15)	321.62(307.51,333.26)	169001.02(156328.66,178330.82)	390.23(360.97,411.78)	255.56(233.78,274.94)	21.33(13.90,27.95)	0.28(-0.03,0.59)
Brunei Darussalam	73.17(53.98,94.16)	467.37(344.75,601.43)	205.43(171.26,245.69)	343.10(286.04,410.33)	180.74(103.19,301.39)	-26.59(-46.87,4.96)	-0.84(-0.97,-0.71)
Bulgaria	3919.98(3440.67,4380.46)	173.20(152.02,193.54)	6474.38(5179.08,8082.34)	272.39(217.89,340.04)	65.16(26.81,118.30)	57.27(20.75,107.86)	1.21(0.89,1.52)
Burkina Faso	1079.30(570.67,2252.04)	149.23(78.91,311.38)	2641.05(1251.25,5244.76)	175.67(83.23,348.86)	144.70(72.52,244.99)	17.72(-17.01,65.96)	0.64(0.58,0.70)
Burundi	846.88(462.07,1399.43)	225.02(122.78,371.84)	925.92(406.08,1762.96)	115.66(50.73,220.22)	9.33(-47.69,80.92)	-48.60(-75.41,-14.94)	-2.74(-3.09,-2.39)
Cabo Verde	71.30(28.35,146.91)	180.65(71.82,372.21)	161.16(77.91,287.65)	210.26(101.65,375.29)	126.03(59.04,258.55)	16.39(-18.10,84.64)	0.11(-0.17,0.38)
Cambodia	340.84(196.69,537.39)	46.00(26.55,72.53)	1247.06(794.38,1991.78)	57.37(36.54,91.63)	265.88(142.32,487.75)	24.71(-17.41,100.33)	0.76(0.69,0.83)
Cameroon	1517.18(982.77,2668.40)	204.90(132.73,360.38)	4044.66(2390.19,7039.95)	198.71(117.43,345.86)	166.59(79.79,298.57)	-3.02(-34.60,44.99)	-0.43(-0.61,-0.25)
Canada	35968.44(33229.80,39109.48)	658.88(608.71,716.42)	25658.05(22824.13,27896.86)	209.09(186.00,227.34)	-28.67(-36.38,-20.56)	-68.27(-71.70,-64.66)	-4.51(-4.84,-4.18)
Central African Republic	450.79(255.10,792.20)	238.75(135.11,419.57)	653.59(344.08,1194.49)	182.72(96.19,333.94)	44.99(0.98,96.34)	-23.47(-46.70,3.64)	-1.12(-1.22,-1.02)
Chad	605.36(281.63,1275.18)	130.63(60.77,275.18)	1216.09(517.66,2420.66)	130.97(55.75,260.70)	100.89(21.46,208.22)	0.26(-39.38,53.83)	-0.24(-0.38,-0.11)
Chile	4381.84(4138.46,4636.19)	260.56(246.09,275.68)	9438.16(8655.00,10147.61)	210.78(193.29,226.63)	115.39(95.90,135.08)	-19.10(-26.43,-11.71)	-0.83(-1.16,-0.50)
China	36486.89(28744.80,47243.64)	25.42(20.03,32.92)	136337.08(105147.11,176012.76)	35.98(27.75,46.45)	273.66(145.54,453.19)	41.51(-7.01,109.51)	1.33(1.19,1.48)
Colombia	9699.07(9097.33,10338.39)	336.89(315.99,359.10)	27050.21(22202.73,32496.71)	282.85(232.16,339.80)	178.89(130.66,236.62)	-16.04(-30.56,1.33)	-1.80(-2.23,-1.37)
Comoros	67.40(25.44,124.62)	207.67(78.39,383.99)	136.42(51.49,252.31)	168.78(63.70,312.15)	102.41(29.32,205.34)	-18.73(-48.08,22.60)	-1.00(-1.20,-0.79)
Congo	648.08(411.98,962.85)	364.06(231.44,540.89)	1251.71(746.22,1926.76)	288.06(171.73,443.41)	93.14(34.61,173.22)	-20.88(-44.86,11.93)	-1.09(-1.27,-0.92)
Cook Islands	6.04(4.80,7.36)	282.82(225.01,344.82)	11.70(6.65,20.16)	248.34(141.01,427.83)	93.93(12.19,258.69)	-12.19(-49.20,62.41)	-0.47(-0.53,-0.41)
Costa Rica	639.83(582.11,713.34)	224.98(204.68,250.83)	2161.41(1886.67,2449.16)	225.74(197.05,255.80)	237.81(182.48,295.92)	0.34(-16.09,17.60)	-0.58(-0.86,-0.30)
Croatia	1330.00(620.33,2371.46)	207.51(96.79,370.00)	3637.91(1607.52,6496.72)	202.87(89.65,362.30)	95.39(52.79,144.92)	45.84(14.05,82.82)	-0.56(-0.79,-0.33)
Cuba	2729.92(2357.64,3180.58)	245.24(211.79,285.72)	5333.88(4511.64,6246.18)	357.66(302.53,418.83)	33.17(14.54,55.66)	-33.95(-43.19,-22.80)	1.04(0.69,1.40)
Cyprus	9290.83(8559.97,9994.30)	548.23(505.10,589.74)	12373.00(10481.30,14016.77)	362.10(306.74,410.20)	54.13(5.67,132.26)	-38.40(-57.77,-7.17)	-1.60(-1.80,-1.40)

Czechia	837.86(650.43,1069.19)	599.80(465.62,765.40)	1291.44(969.14,1622.35)	369.51(277.29,464.19)	46.91(24.73,70.51)	-0.42(-15.46,15.57)	-2.10(-2.37,-1.83)
C 么 te d'Ivoire	7255.74(6735.30,7799.95)	306.43(284.45,329.41)	10659.60(9223.44,12082.91)	305.13(264.02,345.87)	173.53(88.41,284.61)	-2.23(-32.66,37.47)	-0.32(-0.66,0.03)
Democratic People's Republic of Korea	1130.02(804.74,1515.39)	42.46(30.24,56.94)	2459.25(1816.33,3197.03)	43.64(32.23,56.73)	117.63(53.02,213.23)	2.77(-27.74,47.91)	0.35(0.22,0.47)
Democratic Republic of the Congo	5855.67(2895.29,10583.79)	221.74(109.64,400.79)	11180.27(5526.22,19997.86)	185.11(91.50,331.10)	90.93(34.94,168.23)	-16.52(-41.00,17.28)	-0.84(-1.10,-0.58)
Denmark	9966.62(9151.76,10738.29)	763.94(701.48,823.09)	9152.30(8197.59,9940.56)	475.39(425.80,516.33)	-8.17(-18.42,2.54)	-37.77(-44.72,-30.51)	-2.18(-2.48,-1.88)
Djibouti	38.80(19.99,61.67)	180.85(93.17,287.46)	161.62(69.43,271.27)	155.85(66.95,261.58)	316.53(159.06,546.51)	-13.83(-46.41,33.75)	-0.78(-0.94,-0.62)
Dominica	37.36(27.90,47.63)	380.51(284.18,485.11)	52.86(42.31,67.35)	350.44(280.49,446.50)	41.48(-2.72,110.44)	-7.90(-36.68,36.98)	-0.69(-0.95,-0.43)
Dominican Republic	751.86(591.04,973.50)	122.97(96.67,159.23)	2571.87(1848.11,3388.13)	153.91(110.60,202.76)	242.07(134.30,396.95)	25.16(-14.27,81.82)	0.50(0.29,0.72)
Ecuador	1014.15(932.70,1100.55)	117.95(108.47,127.99)	3192.73(2488.66,4094.11)	115.36(89.92,147.93)	214.82(141.28,314.38)	-2.19(-25.04,28.74)	0.34(-0.05,0.72)
Egypt	1367.56(947.42,2042.24)	30.58(21.19,45.67)	4146.34(3210.85,5308.62)	37.47(29.02,47.97)	203.19(87.45,365.83)	22.51(-24.26,88.23)	0.69(0.60,0.77)
El Salvador	243.99(201.42,290.68)	49.90(41.19,59.45)	530.47(409.60,685.84)	51.88(40.06,67.07)	117.41(53.21,195.68)	3.96(-26.74,41.38)	-0.11(-0.27,0.05)
Equatorial Guinea	79.80(44.35,131.41)	250.00(138.93,411.69)	232.79(110.75,402.04)	294.31(140.02,508.29)	191.71(46.40,439.40)	17.73(-40.92,117.69)	0.52(0.43,0.61)
Eritrea	276.04(142.13,507.09)	156.20(80.42,286.95)	658.04(296.08,1230.92)	149.35(67.20,279.37)	138.38(38.10,315.71)	-4.39(-44.61,66.74)	-0.53(-0.68,-0.37)
Estonia	1011.59(919.92,1114.74)	282.19(256.62,310.97)	1559.40(1354.10,1779.40)	356.45(309.53,406.74)	54.15(30.59,82.76)	26.32(7.00,49.76)	0.30(-0.03,0.63)
Eswatini	87.81(58.17,127.95)	194.46(128.83,283.36)	163.99(108.01,234.18)	181.13(119.29,258.65)	86.76(36.00,165.78)	-6.86(-32.17,32.55)	-0.22(-0.43,0.00)
Ethiopia	3375.46(1968.08,6141.67)	105.06(61.25,191.15)	6615.56(3373.42,11346.26)	96.64(49.28,165.75)	95.99(24.85,223.01)	-8.01(-41.40,51.61)	-0.54(-0.78,-0.30)
Fiji	156.57(123.96,191.38)	277.78(219.92,339.53)	371.79(274.59,487.58)	271.89(200.81,356.57)	137.46(65.98,246.87)	-2.12(-31.58,42.98)	-0.29(-0.41,-0.18)
Finland	9943.54(9254.32,10600.90)	837.45(779.41,892.82)	7842.73(6952.38,8506.82)	389.67(345.43,422.66)	-21.13(-28.84,-12.75)	-53.47(-58.02,-48.53)	-2.55(-2.73,-2.38)
France	50824.94(47641.60,53892.04)	365.21(342.34,387.25)	45363.12(40063.92,49021.86)	205.17(181.20,221.72)	-10.75(-19.59,-2.34)	-43.82(-49.39,-38.53)	-2.48(-2.79,-2.17)
Gabon	393.39(245.15,558.96)	401.82(250.40,570.94)	587.61(357.56,868.55)	328.73(200.03,485.90)	49.37(-1.46,114.34)	-18.19(-46.03,17.39)	-0.92(-1.06,-0.77)
Gambia	111.28(45.75,220.07)	200.94(82.62,397.40)	339.75(155.36,595.98)	219.70(100.47,385.39)	205.32(116.42,347.89)	9.34(-22.50,60.39)	0.06(-0.12,0.23)
Georgia	655.61(549.26,789.07)	59.02(49.45,71.04)	3088.43(2583.50,3633.69)	294.17(246.08,346.11)	371.08(250.92,511.61)	398.39(271.27,547.07)	7.47(6.15,8.82)
Germany	71699.61(65797.82,76898.15)	341.70(313.57,366.47)	73648.63(65923.92,79177.39)	233.70(209.19,251.25)	2.72(-7.71,13.83)	-31.61(-38.55,-24.21)	-1.28(-1.39,-1.17)
Ghana	2723.61(1327.08,4563.76)	266.71(129.95,446.90)	6716.46(3042.90,11208.03)	246.28(111.58,410.98)	146.60(65.47,258.51)	-7.66(-38.04,34.25)	-0.57(-0.76,-0.38)
Greece	9772.05(9088.26,10422.53)	365.49(339.92,389.82)	16292.29(14800.86,17403.08)	443.27(402.70,473.50)	66.72(50.86,83.16)	21.28(9.74,33.23)	0.12(-0.15,0.40)

Greenland	18.12(15.66,20.54)	328.90(284.19,372.72)	20.55(16.14,26.57)	151.38(118.90,195.72)	13.40(-12.30,46.77)	-53.97(-64.41,-40.43)	-2.57(-2.73,-2.42)
Grenada	61.24(52.98,73.25)	507.82(439.34,607.40)	84.86(73.37,95.70)	422.48(365.24,476.41)	38.58(11.32,68.76)	-16.80(-33.17,1.31)	-1.56(-2.04,-1.07)
Guam	66.46(56.18,78.20)	515.45(435.72,606.52)	62.42(52.82,73.24)	165.62(140.16,194.34)	-6.08(-26.10,22.01)	-67.87(-74.71,-58.26)	-3.76(-3.94,-3.58)
Guatemala	279.80(252.49,309.65)	48.65(43.90,53.84)	705.19(594.08,841.11)	38.52(32.45,45.94)	152.04(106.20,208.56)	-20.83(-35.23,-3.08)	-1.34(-1.61,-1.06)
Guinea	871.68(420.40,1670.80)	157.52(75.97,301.92)	1546.28(571.39,3023.38)	169.26(62.55,330.95)	77.39(0.28,163.71)	7.46(-39.26,59.74)	-0.03(-0.21,0.14)
Guinea-Bissau	145.72(77.96,264.49)	228.28(122.13,414.35)	215.87(114.73,389.81)	187.92(99.87,339.35)	48.14(3.85,116.55)	-17.68(-42.29,20.34)	-0.87(-0.98,-0.76)
Guyana	118.23(104.89,131.64)	192.88(171.11,214.76)	361.66(275.74,462.47)	321.14(244.84,410.66)	205.89(126.54,294.84)	66.49(23.31,114.91)	1.09(0.38,1.81)
Haiti	1119.97(717.19,1746.83)	207.65(132.97,323.87)	2348.98(1433.77,3685.83)	198.21(120.98,311.02)	109.74(48.07,195.98)	-4.55(-32.61,34.70)	-0.18(-0.27,-0.08)
Honduras	237.07(174.99,330.23)	71.12(52.50,99.07)	1145.94(807.64,1575.38)	108.96(76.80,149.80)	383.38(231.37,649.59)	53.20(5.02,137.58)	1.54(1.40,1.68)
Hungary	7933.41(7300.95,8583.71)	308.01(283.45,333.26)	9951.56(8777.89,11203.75)	312.93(276.02,352.31)	25.44(8.96,42.75)	1.60(-11.75,15.62)	-0.33(-0.48,-0.18)
Iceland	209.64(187.98,227.00)	439.36(393.97,475.73)	243.77(210.05,273.19)	250.31(215.69,280.52)	16.28(2.58,32.60)	-43.03(-49.74,-35.03)	-2.41(-2.79,-2.03)
India	46761.40(27838.63,77331.29)	60.99(36.31,100.86)	222689.03(158767.07,326391.51)	110.77(78.97,162.35)	376.22(254.08,575.37)	81.61(35.03,157.55)	2.01(1.87,2.15)
Indonesia	8148.14(5488.55,10985.70)	50.44(33.97,68.00)	30671.96(20556.48,41544.06)	73.24(49.08,99.20)	276.43(139.50,441.17)	45.21(-7.61,108.76)	1.02(0.85,1.20)
Iran (Islamic Republic of)	1021.08(815.38,1297.28)	22.15(17.69,28.14)	5864.75(5147.07,6627.64)	45.14(39.62,51.02)	474.36(315.09,656.96)	103.82(47.30,168.61)	3.15(2.73,3.58)
Iraq	342.50(242.51,477.31)	27.13(19.21,37.81)	1358.17(945.59,1794.82)	34.96(24.34,46.20)	296.54(139.21,562.53)	28.83(-22.28,115.25)	0.65(0.56,0.75)
Ireland	4550.05(4259.03,4868.82)	668.41(625.66,715.24)	3620.32(3112.88,4016.78)	275.86(237.20,306.07)	-20.43(-30.70,-10.64)	-58.73(-64.05,-53.65)	-3.40(-3.75,-3.04)
Israel	1863.48(1718.89,2002.58)	229.90(212.07,247.07)	2598.35(2315.52,2822.60)	130.01(115.86,141.23)	39.44(24.19,57.78)	-43.45(-49.63,-36.01)	-2.38(-2.63,-2.13)
Italy	48841.61(46473.44,50571.42)	320.92(305.36,332.29)	53536.25(47925.89,57360.68)	235.67(210.97,252.50)	9.61(1.67,16.34)	-26.57(-31.89,-22.06)	-1.60(-1.94,-1.27)
Jamaica	533.95(482.33,601.05)	181.29(163.76,204.07)	907.04(702.73,1156.73)	171.50(132.87,218.71)	69.87(25.66,121.04)	-5.40(-30.02,23.09)	-0.42(-0.77,-0.06)
Japan	79827.81(75025.90,82667.11)	269.57(253.35,279.16)	310349.16(260095.38,337781.04)	594.53(498.26,647.08)	288.77(244.89,313.54)	120.55(95.65,134.60)	2.72(2.63,2.82)
Jordan	219.22(163.02,293.67)	101.96(75.82,136.58)	1030.23(768.19,1347.20)	81.94(61.10,107.15)	369.95(194.15,650.88)	-19.63(-49.69,28.42)	-0.80(-1.09,-0.50)
Kazakhstan	3023.17(2439.01,3856.65)	144.56(116.62,184.41)	6971.15(5577.06,8511.72)	219.67(175.74,268.22)	130.59(65.39,223.69)	51.96(9.00,113.32)	0.44(0.04,0.84)
Kenya	1641.66(1043.45,2385.62)	123.18(78.29,179.00)	5503.44(3238.30,7712.61)	147.12(86.57,206.18)	235.24(142.06,331.59)	19.44(-13.76,53.77)	0.41(0.34,0.48)
Kiribati	2.13(1.73,2.60)	35.70(29.05,43.56)	4.54(3.24,6.17)	36.10(25.75,49.03)	113.46(41.45,202.93)	1.12(-32.99,43.50)	-0.12(-0.21,-0.03)
Kuwait	81.30(72.14,90.73)	88.91(78.89,99.23)	319.84(260.21,397.84)	68.60(55.81,85.33)	293.42(205.73,404.53)	-22.84(-40.04,-1.05)	-0.34(-1.33,0.67)

Kyrgyzstan	132.49(113.00,155.50)	25.73(21.95,30.20)	598.29(470.29,740.84)	70.74(55.61,87.60)	351.57(227.28,501.28)	174.93(99.25,266.08)	3.79(3.19,4.40)
Lao People's Democratic Republic	223.63(139.88,352.31)	65.21(40.79,102.73)	538.86(389.95,746.52)	69.70(50.44,96.56)	140.96(59.05,291.50)	6.88(-29.45,73.65)	0.18(0.15,0.21)
Latvia	1399.32(1283.79,1533.01)	223.81(205.33,245.19)	2092.95(1807.10,2378.09)	318.07(274.63,361.40)	49.57(27.62,75.04)	42.12(21.26,66.32)	0.67(0.36,0.97)
Lebanon	985.81(489.06,1777.73)	260.02(128.99,468.89)	2050.89(1652.17,2596.95)	209.27(168.58,264.99)	108.04(7.01,349.87)	-19.52(-58.60,74.04)	-0.55(-0.82,-0.29)
Lesotho	186.97(103.81,309.42)	133.71(74.24,221.27)	289.02(150.64,471.77)	160.07(83.43,261.29)	54.58(5.45,129.44)	19.72(-18.33,77.70)	0.74(0.58,0.90)
Liberia	383.75(198.51,705.97)	203.65(105.34,374.64)	533.05(229.64,1036.90)	161.47(69.56,314.10)	38.91(-8.53,95.37)	-20.71(-47.79,11.52)	-0.89(-1.00,-0.77)
Libya	30.91(21.45,45.96)	9.94(6.89,14.78)	236.43(145.96,378.56)	28.27(17.45,45.26)	664.78(387.36,1108.32)	184.43(81.25,349.38)	4.14(3.66,4.61)
Lithuania	1640.22(1497.08,1811.98)	206.98(188.92,228.66)	3118.27(2696.75,3556.88)	322.21(278.65,367.53)	90.11(62.36,122.07)	55.67(32.94,81.84)	1.27(1.05,1.48)
Luxembourg	401.72(374.14,433.57)	430.17(400.63,464.28)	388.09(345.28,430.62)	218.40(194.30,242.33)	-3.39(-14.09,9.31)	-49.23(-54.85,-42.55)	-2.73(-3.02,-2.44)
Madagascar	2305.14(1137.25,4022.49)	272.83(134.60,476.09)	3656.68(1798.89,6130.58)	198.01(97.41,331.98)	58.63(11.82,127.31)	-27.42(-48.84,4.00)	-1.27(-1.47,-1.08)
Malawi	817.80(389.87,1587.19)	130.02(61.98,252.34)	1934.66(922.63,3371.20)	165.48(78.92,288.35)	136.57(68.96,234.18)	27.27(-9.10,79.78)	0.44(0.26,0.63)
Malaysia	4383.32(3514.72,5376.85)	297.00(238.14,364.31)	16031.03(13485.64,19120.59)	326.98(275.06,389.99)	265.73(165.71,404.15)	10.10(-20.01,51.76)	-0.12(-0.37,0.12)
Maldives	7.42(5.12,10.53)	48.42(33.41,68.69)	23.52(11.07,39.11)	43.23(20.34,71.90)	216.84(73.83,434.03)	-10.71(-51.01,50.50)	-0.76(-1.00,-0.53)
Mali	694.19(349.58,1424.71)	103.82(52.28,213.07)	1443.05(604.49,2878.36)	99.70(41.76,198.87)	107.88(17.14,251.00)	-3.97(-45.88,62.16)	-0.08(-0.27,0.11)
Malta	189.14(174.96,205.33)	261.08(241.51,283.43)	223.46(198.87,249.64)	141.61(126.02,158.20)	18.14(3.58,34.63)	-45.76(-52.45,-38.19)	-2.75(-3.10,-2.39)
Marshall Islands	6.31(4.15,9.23)	247.41(162.90,362.02)	12.48(8.18,17.99)	213.07(139.67,306.99)	97.84(43.92,176.52)	-13.88(-37.35,20.37)	-0.78(-0.87,-0.69)
Mauritania	420.55(192.33,678.70)	253.33(115.85,408.83)	741.61(275.30,1307.78)	206.72(76.74,364.53)	76.34(3.02,171.27)	-18.40(-52.33,25.53)	-1.15(-1.35,-0.95)
Mauritius	150.14(140.38,159.90)	123.43(115.41,131.46)	242.24(222.22,258.74)	71.60(65.68,76.47)	61.35(46.70,74.91)	-41.99(-47.26,-37.12)	-1.92(-2.37,-1.47)
Mexico	4457.25(4320.23,4569.97)	64.11(62.14,65.73)	11941.18(10468.10,13552.46)	55.41(48.57,62.89)	167.90(135.28,205.09)	-13.57(-24.09,-1.57)	-0.92(-1.12,-0.72)
Micronesia (Federated States of)	24.95(17.89,35.11)	313.35(224.76,441.01)	32.04(22.78,43.10)	243.44(173.10,327.51)	28.42(-6.78,87.44)	-22.31(-43.60,13.40)	-1.12(-1.21,-1.02)
Monaco	68.51(50.78,86.28)	618.23(458.19,778.54)	84.57(63.74,114.20)	540.51(407.38,729.90)	23.43(-12.51,83.59)	-12.57(-38.03,30.04)	-0.46(-0.64,-0.29)
Mongolia	52.81(36.91,73.55)	30.48(21.30,42.44)	233.17(174.97,298.85)	59.06(44.32,75.69)	341.49(184.92,592.31)	93.77(25.05,203.86)	2.42(2.10,2.74)
Montenegro	678.72(526.94,889.71)	623.01(483.69,816.69)	1429.98(1065.86,1882.37)	820.07(611.25,1079.51)	110.69(48.44,210.06)	31.63(-7.26,93.71)	1.18(1.08,1.27)
Morocco	264.72(173.33,366.04)	11.29(7.39,15.61)	2323.66(1592.92,3041.84)	38.68(26.51,50.63)	777.77(520.37,1102.44)	242.58(142.12,369.30)	4.75(4.47,5.03)
Mozambique	1560.59(672.48,3154.37)	159.70(68.82,322.80)	3749.41(1530.17,7307.35)	209.57(85.53,408.43)	140.26(63.26,232.31)	31.22(-10.83,81.50)	0.99(0.93,1.05)

Myanmar	2554.18(1473.26,3798.30)	65.25(37.64,97.03)	6315.21(4755.68,8381.48)	74.81(56.33,99.28)	147.25(62.41,300.46)	14.65(-24.69,85.69)	0.32(0.20,0.43)
Namibia	220.47(142.61,336.96)	203.18(131.43,310.54)	439.04(315.03,606.16)	194.31(139.43,268.27)	99.13(40.36,193.43)	-4.37(-32.59,40.92)	-0.43(-0.63,-0.24)
Nauru	3.11(2.34,4.07)	430.32(323.48,563.10)	3.80(2.58,5.15)	411.61(279.56,557.72)	22.32(-13.94,70.66)	-4.35(-32.70,33.45)	-0.34(-0.52,-0.17)
Nepal	839.75(448.54,1525.14)	54.55(29.14,99.08)	4021.87(2727.63,6302.31)	101.62(68.92,159.24)	378.93(210.38,654.51)	86.27(20.71,193.45)	2.25(2.05,2.44)
Netherlands	25959.58(24110.12,27810.82)	784.05(728.19,839.96)	20925.19(18315.59,22691.96)	357.64(313.04,387.84)	-19.39(-27.08,-11.48)	-54.39(-58.73,-49.91)	-3.28(-3.67,-2.90)
New Zealand	6880.14(6417.71,7329.81)	1045.66(975.37,1114.00)	5414.75(4844.04,5842.39)	379.60(339.59,409.58)	-21.30(-29.10,-14.36)	-63.70(-67.30,-60.50)	-3.95(-4.18,-3.72)
Nicaragua	78.65(65.49,93.85)	31.65(26.36,37.77)	265.23(207.84,334.68)	32.58(25.53,41.11)	237.25(147.90,346.95)	2.94(-24.33,36.43)	0.02(-0.25,0.29)
Niger	484.42(204.93,1032.60)	110.22(46.63,234.95)	1304.05(481.68,3019.10)	93.67(34.60,216.86)	169.19(71.53,282.16)	-15.02(-45.85,20.64)	-0.75(-0.91,-0.60)
Nigeria	15787.20(7876.40,29533.65)	216.71(108.12,405.40)	26317.64(12027.71,48315.50)	175.43(80.18,322.07)	66.70(13.89,133.16)	-19.05(-44.69,13.23)	-1.03(-1.21,-0.85)
Niue	1.28(1.02,1.59)	351.81(279.90,435.28)	1.06(0.80,1.33)	269.82(205.51,338.89)	-17.60(-38.47,7.98)	-23.31(-42.73,0.50)	-0.93(-0.95,-0.90)
North Macedonia	564.89(441.20,707.47)	171.72(134.12,215.06)	1450.33(909.18,2218.28)	244.61(153.34,374.14)	156.75(55.01,299.86)	42.45(-13.99,121.86)	0.90(0.70,1.10)
Northern Mariana Islands	9.43(7.43,12.05)	386.13(304.19,493.43)	23.74(18.54,30.80)	244.19(190.74,316.89)	151.79(75.73,267.23)	-36.76(-55.86,-7.77)	-2.70(-3.09,-2.31)
Norway	9646.36(9155.18,10039.01)	890.77(845.41,927.03)	7961.07(7071.41,8488.72)	490.90(436.04,523.43)	-17.47(-22.90,-12.84)	-44.89(-48.51,-41.80)	-2.67(-3.01,-2.34)
Oman	16.82(10.24,27.60)	16.73(10.19,27.46)	161.36(89.19,275.94)	51.66(28.55,88.34)	859.56(258.95,2281.62)	208.76(15.50,666.35)	4.27(3.80,4.74)
Pakistan	7181.97(4858.89,10950.73)	78.75(53.28,120.08)	23960.76(17432.71,33196.77)	120.86(87.93,167.44)	233.62(137.71,365.25)	53.46(9.34,114.01)	1.13(0.85,1.41)
Palau	4.25(3.19,5.43)	265.70(199.47,339.16)	8.34(6.29,11.22)	199.26(150.17,267.85)	96.24(36.90,186.61)	-25.01(-47.68,9.53)	-1.15(-1.24,-1.06)
Palestine	81.27(58.22,111.92)	55.99(40.11,77.11)	283.03(215.93,359.30)	65.78(50.19,83.51)	248.28(122.39,447.98)	17.49(-24.98,84.85)	0.63(0.51,0.75)
Panama	536.41(486.68,584.85)	221.39(200.87,241.38)	1276.53(965.98,1559.62)	171.68(129.91,209.75)	137.97(81.80,192.28)	-22.46(-40.76,-4.76)	-1.34(-1.58,-1.10)
Papua New Guinea	367.28(231.06,582.00)	124.13(78.09,196.71)	1020.94(659.91,1524.02)	124.31(80.35,185.56)	177.97(85.40,329.84)	0.14(-33.21,54.85)	-0.21(-0.32,-0.10)
Paraguay	727.50(583.54,902.57)	200.54(160.86,248.80)	2842.13(2159.31,3693.31)	287.05(218.09,373.02)	290.67(179.17,465.40)	43.14(2.29,107.16)	1.18(1.05,1.31)
Peru	1334.65(1018.56,1743.91)	67.69(51.66,88.45)	3338.10(2465.83,4503.13)	59.68(44.08,80.51)	150.11(66.08,266.03)	-11.84(-41.45,29.03)	-0.50(-0.65,-0.35)
Philippines	4332.90(3615.08,5125.02)	90.76(75.72,107.35)	15099.89(12223.85,17853.92)	108.28(87.66,128.03)	248.49(180.79,349.12)	19.31(-3.87,53.75)	0.44(0.34,0.54)
Poland	34785.66(33664.27,35781.72)	451.41(436.86,464.34)	43033.02(38697.43,47583.47)	355.19(319.41,392.75)	23.71(12.05,36.85)	-21.31(-28.73,-12.96)	-1.44(-1.69,-1.20)
Portugal	4056.12(3782.41,4349.66)	166.37(155.15,178.41)	6419.99(5851.36,6907.46)	165.01(150.40,177.54)	58.28(42.36,74.11)	-0.82(-10.80,9.10)	-0.39(-0.58,-0.21)
Puerto Rico	1312.17(1220.80,1403.41)	216.26(201.20,231.29)	1144.53(943.31,1339.72)	98.04(80.81,114.76)	-12.78(-27.33,3.07)	-54.66(-62.23,-46.43)	-3.37(-3.65,-3.09)

Qatar	17.28(13.18,22.67)	105.79(80.71,138.79)	105.39(54.21,196.42)	68.80(35.39,128.22)	509.86(170.98,1247.91)	-34.97(-71.10,43.74)	-1.82(-2.24,-1.39)
Republic of Korea	6846.55(4516.73,9852.18)	137.57(90.76,197.96)	25872.02(20687.08,30999.92)	154.70(123.70,185.36)	277.88(131.82,541.20)	12.45(-31.01,90.81)	0.25(-0.03,0.52)
Republic of Moldova	677.53(603.76,754.44)	87.76(78.21,97.72)	1662.86(1459.39,1881.19)	157.49(138.22,178.17)	145.43(106.09,194.46)	79.45(50.69,115.30)	1.76(1.44,2.08)
Romania	6164.38(5658.66,6712.14)	123.19(113.09,134.14)	12029.87(10428.46,13920.16)	200.32(173.65,231.80)	95.15(65.83,130.19)	62.61(38.18,91.81)	1.22(1.02,1.42)
Russian Federation	82296.31(79962.64,84208.55)	260.74(253.35,266.80)	182953.28(167026.09,199483.27)	430.43(392.95,469.31)	122.31(102.33,142.18)	65.08(50.24,79.84)	1.22(0.81,1.64)
Rwanda	1122.19(656.83,1782.43)	239.81(140.36,380.90)	1579.76(934.28,2641.25)	149.39(88.35,249.77)	40.77(-15.40,134.39)	-37.70(-62.56,3.72)	-2.47(-2.91,-2.04)
Saint Kitts and Nevis	24.74(22.20,27.52)	386.89(347.11,430.35)	25.66(20.69,32.40)	199.24(160.66,251.54)	3.72(-18.26,31.68)	-48.50(-59.42,-34.62)	-2.79(-3.18,-2.39)
Saint Lucia	127.74(116.24,142.54)	873.17(794.56,974.33)	173.98(144.70,204.43)	413.42(343.85,485.79)	36.19(10.20,66.44)	-52.65(-61.69,-42.14)	-3.16(-3.46,-2.85)
Saint Vincent and the Grenadines	29.46(27.09,32.38)	245.19(225.46,269.54)	46.20(40.51,52.58)	181.70(159.31,206.80)	56.83(35.45,86.39)	-25.89(-36.00,-11.92)	-1.59(-1.97,-1.20)
Samoa	39.19(27.87,52.70)	271.95(193.39,365.67)	56.19(42.99,72.94)	228.22(174.59,296.23)	43.37(10.06,94.25)	-16.08(-35.58,13.70)	-0.70(-0.78,-0.62)
San Marino	18.49(14.79,22.91)	314.86(251.88,390.14)	21.60(13.73,33.27)	182.63(116.11,281.31)	16.81(-30.09,85.31)	-42.00(-65.28,-7.98)	-0.68(-1.12,-0.24)
Sao Tome and Principe	17.00(8.23,31.07)	151.84(73.50,277.49)	34.66(17.67,61.57)	189.57(96.64,336.76)	103.86(48.74,175.11)	24.85(-8.91,68.49)	0.73(0.60,0.85)
Saudi Arabia	56.98(38.81,82.29)	6.40(4.36,9.24)	464.57(335.20,638.50)	15.43(11.13,21.21)	715.26(409.83,1237.95)	141.24(50.86,295.92)	3.63(3.16,4.09)
Senegal	1035.01(479.63,1912.71)	195.86(90.76,361.96)	2374.13(997.37,4398.23)	183.87(77.25,340.64)	129.38(63.19,211.91)	-6.12(-33.21,27.66)	-0.51(-0.64,-0.39)
Serbia	7139.20(6014.30,8447.30)	343.27(289.18,406.17)	12127.37(9565.74,15305.84)	432.31(341.00,545.62)	69.87(28.12,128.61)	25.94(-5.01,69.49)	0.71(0.54,0.89)
Seychelles	9.54(7.83,11.53)	99.44(81.68,120.17)	17.17(11.37,24.62)	82.76(54.83,118.67)	79.97(24.54,155.14)	-16.78(-42.41,17.98)	-0.56(-0.85,-0.26)
Sierra Leone	683.40(321.30,1290.65)	206.18(96.93,389.39)	1017.44(438.62,1999.17)	168.43(72.61,330.94)	48.88(1.38,116.24)	-18.31(-44.37,18.65)	-0.87(-0.97,-0.78)
Singapore	917.52(866.09,966.46)	253.06(238.88,266.56)	2788.71(2532.85,2992.03)	183.61(166.76,197.00)	203.94(174.59,231.37)	-27.44(-34.45,-20.90)	-1.12(-1.31,-0.94)
Slovakia	2369.35(2006.47,2816.62)	228.38(193.40,271.49)	3880.45(2925.29,5078.59)	236.57(178.34,309.62)	63.78(13.20,134.62)	3.59(-28.40,48.39)	-0.06(-0.20,0.08)
Slovenia	1159.14(1078.81,1243.19)	269.13(250.47,288.64)	1673.58(1401.58,2011.83)	228.57(191.42,274.77)	44.38(21.45,73.56)	-15.07(-28.56,2.10)	-0.82(-1.18,-0.45)
Solomon Islands	28.57(18.35,48.84)	126.37(81.16,216.02)	76.13(51.02,112.37)	140.35(94.06,207.14)	166.47(82.47,274.32)	11.06(-23.95,56.01)	0.31(0.18,0.44)
Somalia	373.36(196.27,787.56)	103.69(54.51,218.72)	708.92(319.76,1715.53)	75.17(33.91,181.91)	89.88(22.24,193.56)	-27.50(-53.33,12.08)	-1.30(-1.53,-1.06)
South Africa	8246.38(6254.80,10341.77)	244.30(185.30,306.38)	14598.19(12774.64,16441.73)	185.06(161.94,208.43)	77.03(45.10,135.13)	-24.25(-37.91,0.61)	-1.65(-2.09,-1.21)
South Sudan	784.16(305.78,1467.64)	189.09(73.74,353.90)	720.77(307.63,1417.20)	115.75(49.40,227.58)	-8.08(-37.66,35.68)	-38.79(-58.48,-9.64)	-1.98(-2.23,-1.73)
Spain	24224.38(22798.92,25598.55)	253.38(238.47,267.75)	35143.71(31750.49,37722.59)	227.10(205.17,243.76)	45.08(33.03,56.06)	-10.37(-17.81,-3.59)	-1.10(-1.49,-0.71)

Sri Lanka	460.96(379.05,569.08)	25.90(21.30,31.97)	1403.14(924.68,2006.07)	29.13(19.20,41.65)	204.40(85.35,385.43)	12.48(-31.51,79.38)	0.57(0.41,0.74)
Sudan	137.80(73.16,320.80)	9.27(4.92,21.57)	919.70(583.35,1371.39)	29.54(18.73,44.04)	567.41(222.41,1129.98)	218.73(53.97,487.39)	4.40(4.13,4.68)
Suriname	95.98(79.55,114.64)	220.57(182.80,263.43)	213.69(149.09,293.60)	191.30(133.47,262.84)	122.63(46.19,219.66)	-13.27(-43.05,24.53)	-0.55(-0.77,-0.32)
Sweden	20020.45(18784.51,21062.13)	843.29(791.24,887.17)	14339.77(12495.14,16031.36)	425.96(371.16,476.21)	-28.37(-36.14,-20.66)	-49.49(-54.97,-44.05)	-2.75(-3.04,-2.46)
Switzerland	8818.00(8092.05,9542.66)	522.03(479.06,564.93)	6927.60(6046.46,7596.65)	236.37(206.31,259.20)	-21.44(-30.40,-11.63)	-54.72(-59.89,-49.07)	-2.64(-2.81,-2.47)
Syrian Arab Republic	272.97(189.83,380.65)	30.96(21.53,43.17)	865.09(624.96,1187.63)	36.61(26.45,50.27)	216.92(93.96,407.72)	18.26(-27.62,89.46)	0.34(0.24,0.44)
Taiwan (Province of China)	3283.22(3062.19,3500.03)	118.37(110.40,126.19)	15504.76(13926.08,16877.42)	205.91(184.94,224.14)	372.24(322.47,421.58)	73.95(55.62,92.12)	0.76(0.02,1.51)
Tajikistan	118.11(85.14,151.16)	25.22(18.18,32.28)	210.68(150.58,288.52)	20.51(14.66,28.09)	78.37(14.60,172.17)	-18.67(-47.75,24.10)	-0.79(-1.23,-0.34)
Thailand	7810.67(5827.94,10485.44)	131.62(98.21,176.69)	31227.60(24016.64,40561.38)	160.65(123.56,208.67)	299.81(167.13,501.87)	22.06(-18.45,83.75)	0.28(0.13,0.42)
Timor-Leste	17.02(12.20,25.62)	39.16(28.07,58.96)	87.70(57.54,137.49)	61.74(40.51,96.80)	415.25(239.99,652.66)	57.67(4.04,130.31)	1.56(1.41,1.70)
Togo	406.52(197.07,742.67)	208.39(101.02,380.71)	1207.36(502.07,2182.26)	193.27(80.37,349.34)	197.00(102.21,300.87)	-7.25(-36.85,25.18)	-0.59(-0.73,-0.45)
Tokelau	0.70(0.54,0.89)	296.67(227.30,377.18)	0.75(0.50,1.04)	296.48(196.38,411.30)	6.74(-33.10,53.77)	-0.06(-37.36,43.97)	-0.29(-0.52,-0.06)
Tonga	22.94(17.95,29.45)	241.79(189.21,310.40)	37.02(25.95,48.82)	275.63(193.22,363.48)	61.34(2.33,138.34)	13.99(-27.70,68.40)	0.31(0.10,0.53)
Trinidad and Tobago	711.00(655.82,781.33)	517.84(477.65,569.07)	1274.28(973.79,1630.21)	365.82(279.55,467.99)	79.22(33.04,131.24)	-29.36(-47.56,-8.86)	-1.89(-2.27,-1.51)
Tunisia	113.67(78.65,153.97)	13.05(9.03,17.68)	878.18(566.25,1287.81)	37.58(24.23,55.10)	672.57(382.04,1152.72)	187.83(79.59,366.71)	3.84(3.47,4.20)
Turkey	11599.70(8213.18,16710.25)	194.15(137.47,279.69)	35705.49(27769.20,45418.97)	216.12(168.08,274.91)	207.81(90.11,377.43)	11.31(-31.25,72.65)	0.21(0.02,0.41)
Turkmenistan	314.18(259.67,381.84)	96.74(79.95,117.57)	1214.09(897.29,1750.73)	171.55(126.79,247.38)	286.44(153.75,470.28)	77.34(16.45,161.71)	1.69(1.45,1.93)
Tuvalu	2.79(1.82,3.94)	239.77(156.61,339.13)	4.24(3.34,5.42)	232.68(183.50,297.32)	52.21(10.46,120.11)	-2.96(-29.58,40.33)	-0.37(-0.49,-0.25)
Uganda	1462.64(671.00,2819.85)	139.84(64.15,269.61)	3185.62(1517.06,5463.56)	134.33(63.97,230.38)	117.80(44.41,235.14)	-3.94(-36.31,47.81)	-0.46(-0.59,-0.32)
Ukraine	26969.12(24339.36,29973.14)	216.32(195.23,240.42)	33152.28(24364.88,43318.84)	244.16(179.44,319.04)	22.93(-13.30,64.56)	12.87(-20.39,51.10)	-0.04(-0.37,0.30)
United Arab Emirates	66.31(38.68,103.37)	118.15(68.91,184.18)	591.20(459.06,787.02)	82.33(63.93,109.60)	791.57(451.91,1552.64)	-30.31(-56.86,29.17)	0.03(-0.60,0.66)
United Kingdom	158249.66(151615.20,161878.81)	1065.44(1020.77,1089.87)	85116.94(77181.19,89620.41)	404.74(367.00,426.15)	-46.21(-49.42,-44.31)	-62.01(-64.27,-60.67)	-3.78(-4.15,-3.41)
United Republic of Tanzania	3580.28(2134.38,5998.23)	198.44(118.30,332.46)	9067.93(4331.33,15664.07)	219.98(105.07,379.99)	153.27(45.15,345.55)	10.85(-36.47,95.01)	-0.02(-0.16,0.11)
United States of America	291231.59(274421.51,300868.52)	555.13(523.08,573.50)	188420.24(172461.27,197574.71)	187.95(172.04,197.09)	-35.30(-37.61,-33.27)	-66.14(-67.35,-65.08)	-4.35(-4.68,-4.03)
United States Virgin Islands	39.21(31.66,47.03)	280.74(226.66,336.68)	59.00(43.54,75.62)	183.28(135.26,234.91)	50.46(7.00,110.28)	-34.72(-53.57,-8.76)	-1.68(-1.98,-1.37)

Uruguay	4072.67(3783.29,4401.41)	603.56(560.68,652.28)	4204.42(3840.32,4546.71)	464.94(424.68,502.79)	3.24(-7.84,14.87)	-22.97(-31.23,-14.28)	-1.27(-1.57,-0.97)
Uzbekistan	379.93(281.35,529.33)	19.43(14.39,27.07)	3955.82(3138.88,4915.55)	85.10(67.52,105.74)	941.20(593.49,1412.90)	338.03(191.75,536.48)	5.45(4.79,6.11)
Vanuatu	17.56(12.02,26.17)	176.22(120.55,262.54)	48.22(35.61,65.88)	168.15(124.20,229.76)	174.54(101.93,281.86)	-4.58(-29.81,32.73)	-0.48(-0.62,-0.33)
Venezuela (Bolivarian Republic of)	3463.52(3220.24,3753.07)	221.19(205.65,239.68)	8930.58(6918.05,11290.65)	170.69(132.22,215.79)	157.85(94.91,232.36)	-22.83(-41.67,-0.53)	-1.62(-1.99,-1.24)
Viet Nam	3814.25(2712.93,5423.16)	54.71(38.91,77.78)	14416.57(10464.75,19485.79)	82.53(59.91,111.55)	277.97(152.14,495.12)	50.85(0.64,137.52)	1.44(1.17,1.71)
Yemen	75.86(38.81,134.43)	9.46(4.84,16.76)	670.15(388.47,1063.08)	29.80(17.27,47.27)	783.40(453.30,1391.27)	215.04(97.32,431.82)	4.31(3.93,4.69)
Zambia	858.11(543.07,1318.78)	186.41(117.98,286.49)	3171.58(1355.68,5651.29)	293.62(125.51,523.19)	269.60(86.45,579.92)	57.51(-20.54,189.76)	1.61(1.22,2.00)
Zimbabwe	1783.18(1382.67,2184.00)	269.03(208.60,329.50)	3534.25(2622.20,4711.78)	319.19(236.82,425.53)	98.20(34.62,188.67)	18.65(-19.41,72.80)	0.31(0.02,0.60)
